# Supplementary material for: 17-Oxime ethers of oxidized ecdysteroid derivatives modulate oxidative stress in human brain endothelial cells and dose-dependently might protect or damage the blood-brain barrier
Source: PLoS One. 2024 Feb 22;19(2):e0290526. doi: 10.1371/journal.pone.0290526 (PMC10883584; doi:10.1371/journal.pone.0290526)

SUPPORTING INFORMATION

17-Oxime ethers of oxidized ecdysteroid derivatives modulate oxidative stress in human brain endothelial cells and dose-dependently might protect or damage the blood-brain barrier

Máté Vágvölgyi[1,#], Dávid Laczkó[1,#], Ana Raquel Santa-Maria[2,3], Fruzsina R. Walter[2], Róbert Berkecz[4], Mária Deli[2], Gábor Tóth[5], Attila Hunyadi*[,1,6]

1 Institute of Pharmacognosy, University of Szeged, H-6720 Szeged, Hungary, **2** Institute of Biophysics, Biological Research Centre, Szeged, H-6726 Hungary, **3** Wyss Institute for Biologically Inspired Engineering at Harvard University, Boston, MA 02115, USA, **4** Institute of Pharmaceutical Analysis, University of Szeged, H-6720 Hungary, **5** Department of Inorganic and Analytical Chemistry, NMR Group, Budapest University of Technology and Economics, H-1111 Budapest, Hungary, **6** Interdisciplinary Centre of Natural Products, University of Szeged, H-6720 Szeged, Hungary*: corresponding author, email: hunyadi.attila@szte.hu

CONTENTS

	Page
Figure S1. Compound **3** ^{1}H NMR + selROE on CH_3-21	2
Figure S2. Compound **3** DEPTQ	3
Figure S3. Compound **3** edHSQC section + selROE on CH_3-21	4
Figure S4. Compound **3** HMBC and HMBC CH_3 section	5
Figure S5. Compound **4** ^{1}H NMR	6
Figure S6. Compound **4** DEPTQ	7
Figure S7. Compound **4** edHSQC	8
Figure S8. Compound **4** edHSQC section + selROE on CH_3-21	9
Figure S9. Compound **4** HMBC and HMBC CH_3 section	10

	Page
Figure S9. Compound **4** HMBC and HMBC CH_3 section	11
Figure S10. Compound **5** ^{1}H NMR	12
Figure S11. Compound **5** DEPTQ	13
Figure S12. Compound **5** edHSQC	14
Figure S13. Compound **5** edHSQC section + selROE on CH_3-21	15
Figure S14. Compound **5** HMBC	16
Figure S15. Compound **6** ^{1}H NMR + DEPTQ	17
Figure S16. Compound **6** edHSQC	18
Figure S17. Compound **7** ^{1}H NMR + DEPTQ	19
Figure S18. Compound **7** edHSQC	20
Figure S19. Compound **8** ^{1}H NMR + DEPTQ	21
Figure S20. Compound **8** edHSQC	22
Figure S21. HR-MS spectrum of compound **3** recorded in positive ionization mode	23
Figure S22. HR-MS spectrum of compound **4** recorded in positive ionization mode	24
Figure S23. HR-MS spectrum of compound **5** recorded in positive ionization mode	25
Figure S24. HR-MS spectrum of compound **6** recorded in positive ionization mode	26
Figure S25. HR-MS spectrum of compound **7** recorded in positive ionization mode	27
Figure S26. HR-MS spectrum of compound **8** recorded in positive ionization mode	28
Figure S27. The impact of compounds **2**–**8** on the viability of human brain endothelial cells (hCMEC/D3)	29
Figure S28. The effects of compounds **4** and **6** at selected concentrations on human brain microvascular endothelial cells (hCMEC/D3).	30

Figure S1. Compound **3** ^{1}H NMR + selROE on CH_3-21

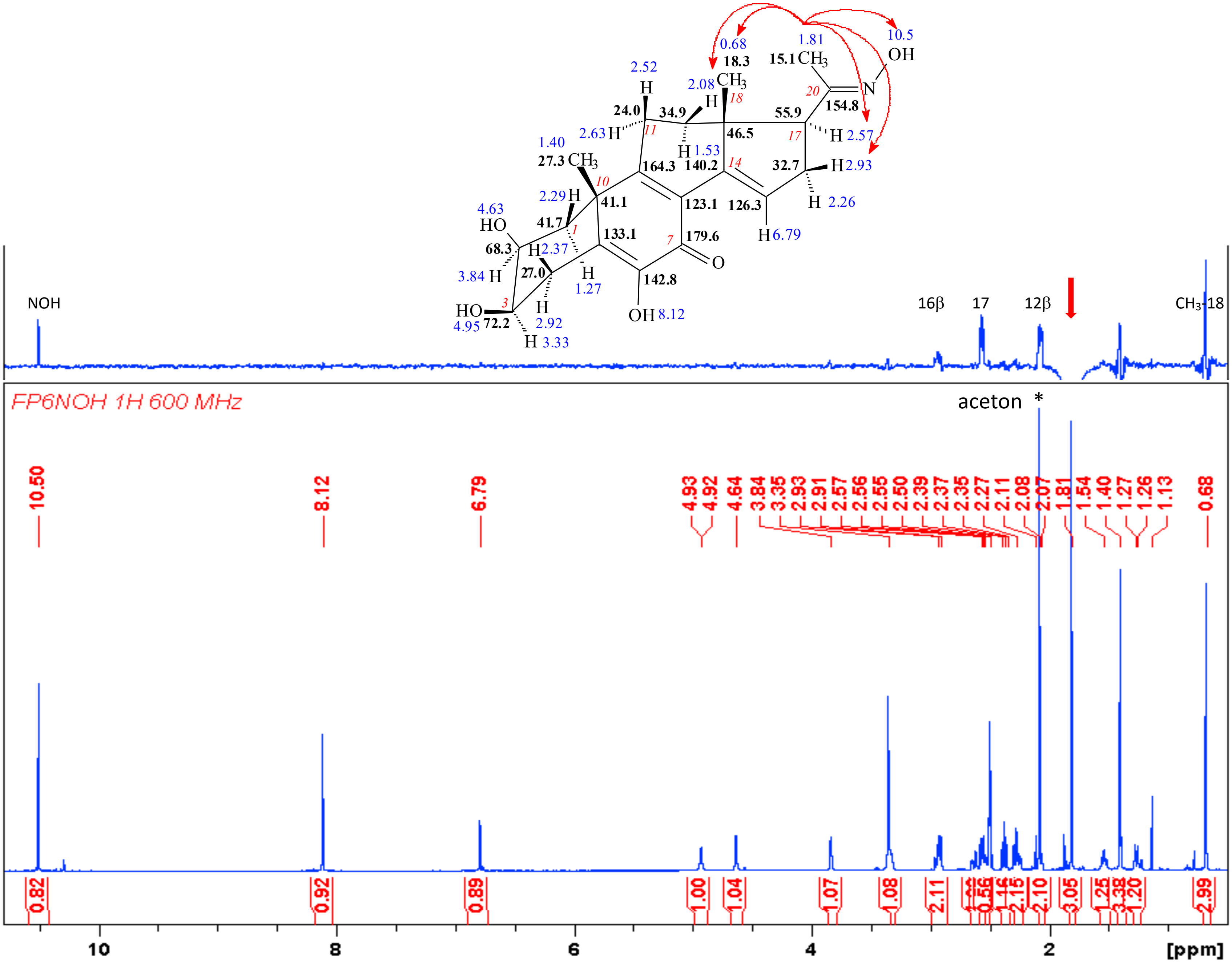

Figure S2. Compound **3** DEPTQ

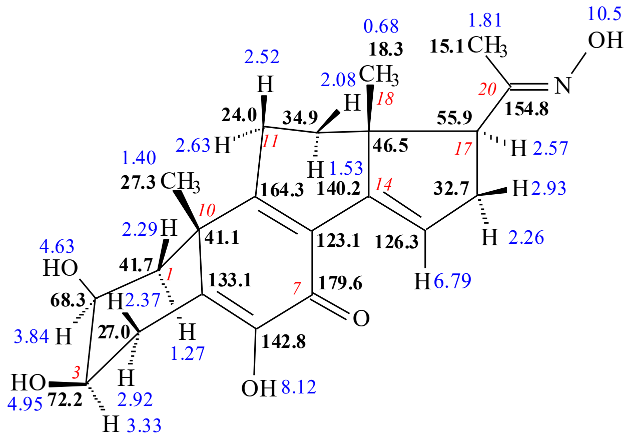

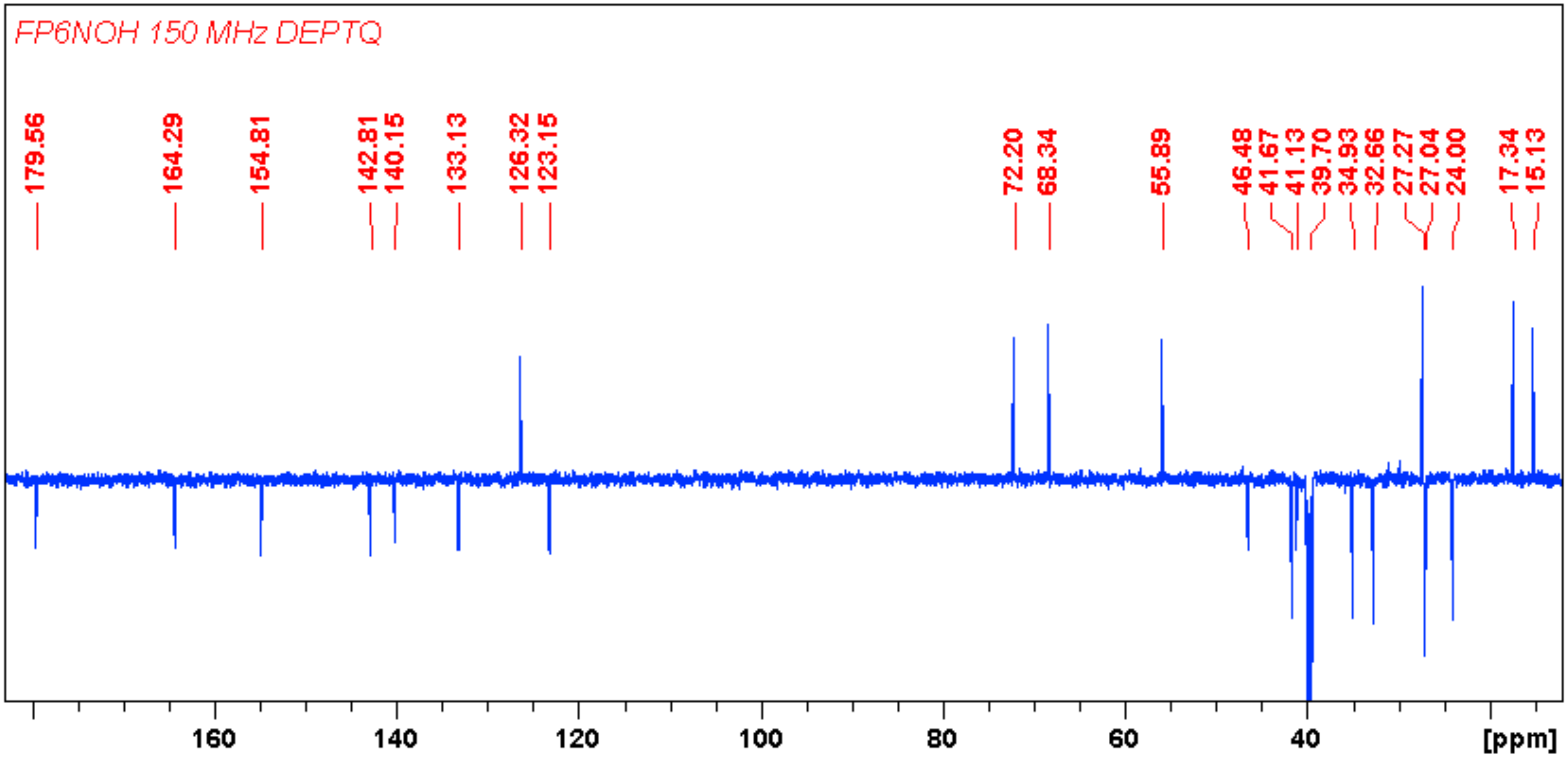

Figure S3. Compound **3** edHSQC section + selROE on CH_3-21

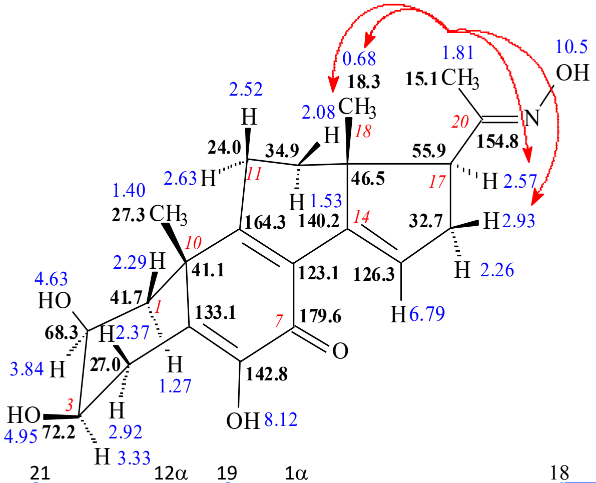

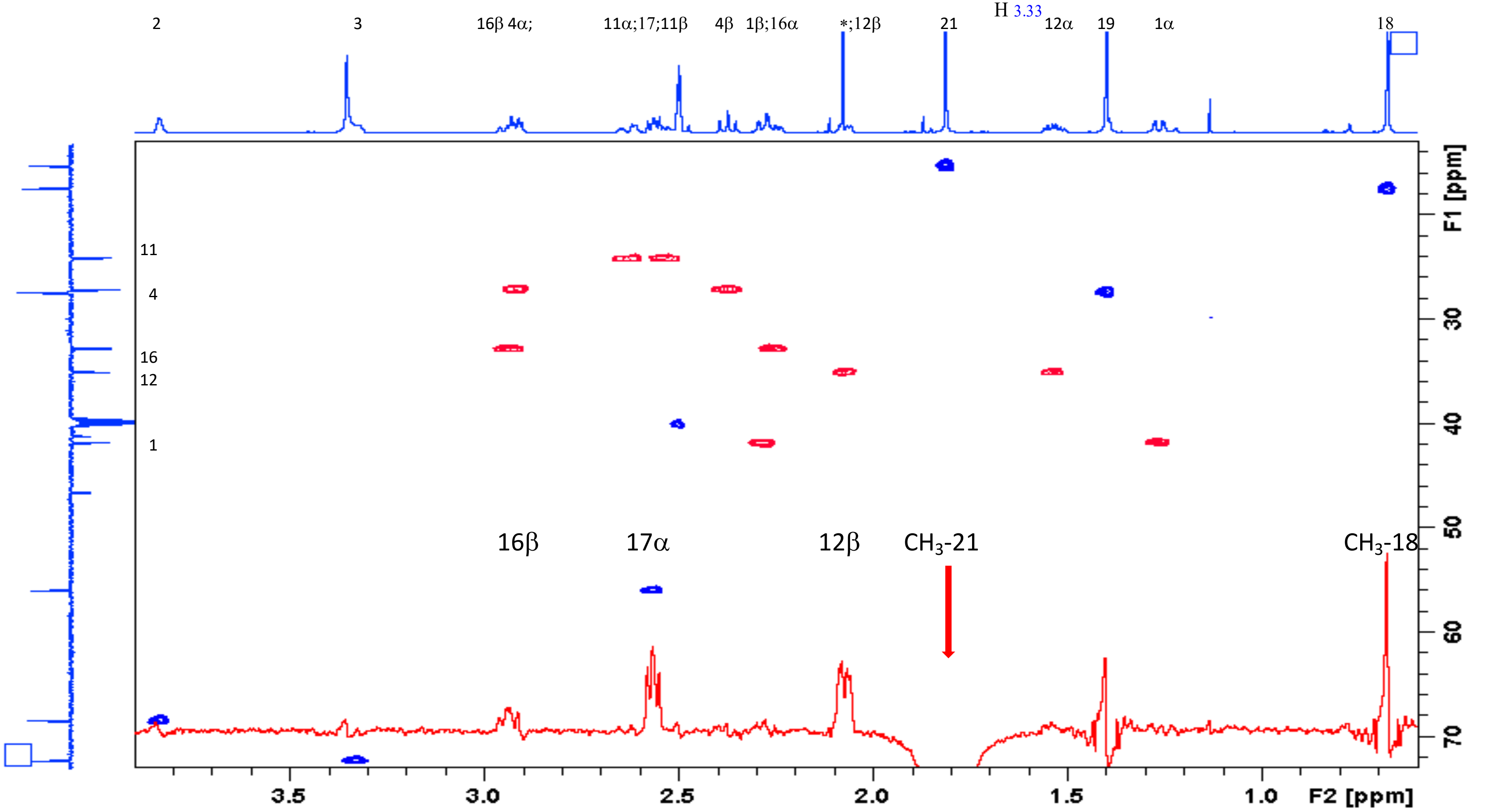

Figure S4. Compound **3** HMBC

HMBC CH_3 section

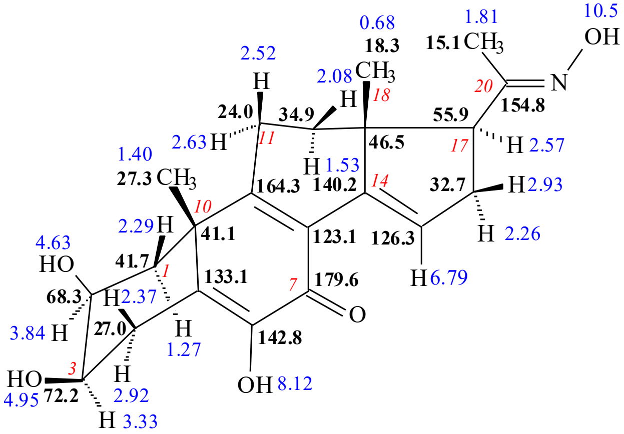

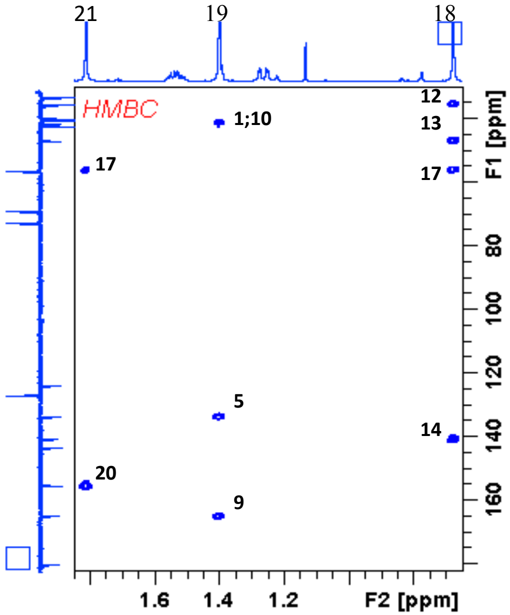

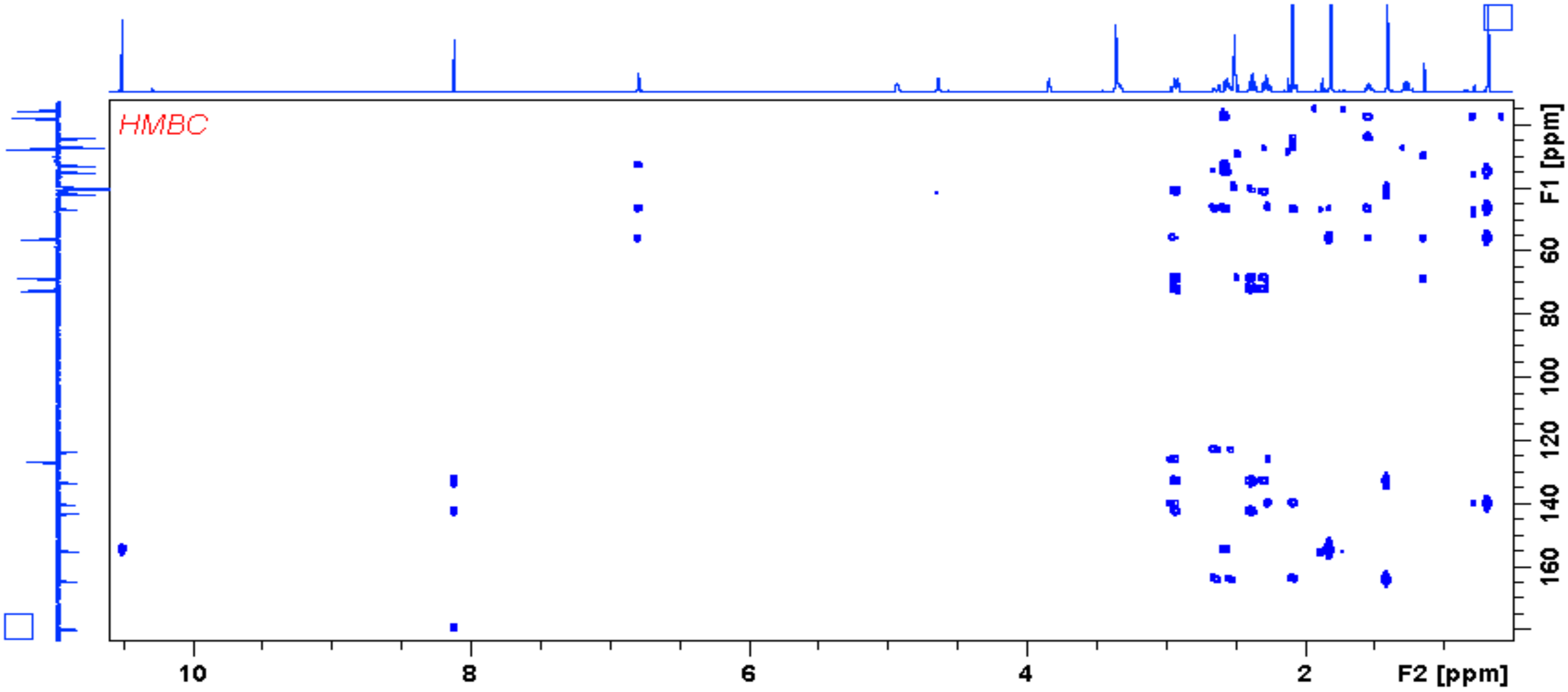

Figure S5. Compound **4** ^{1}H NMR

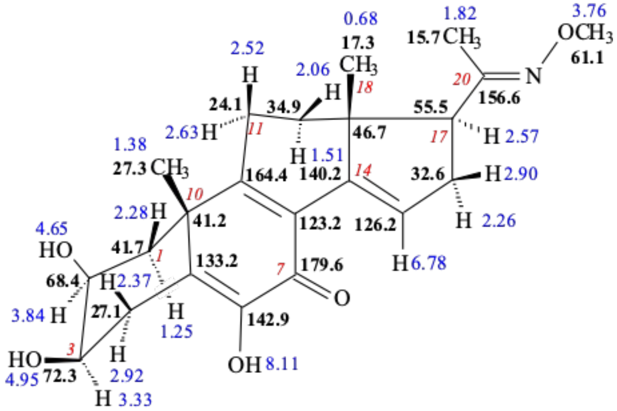

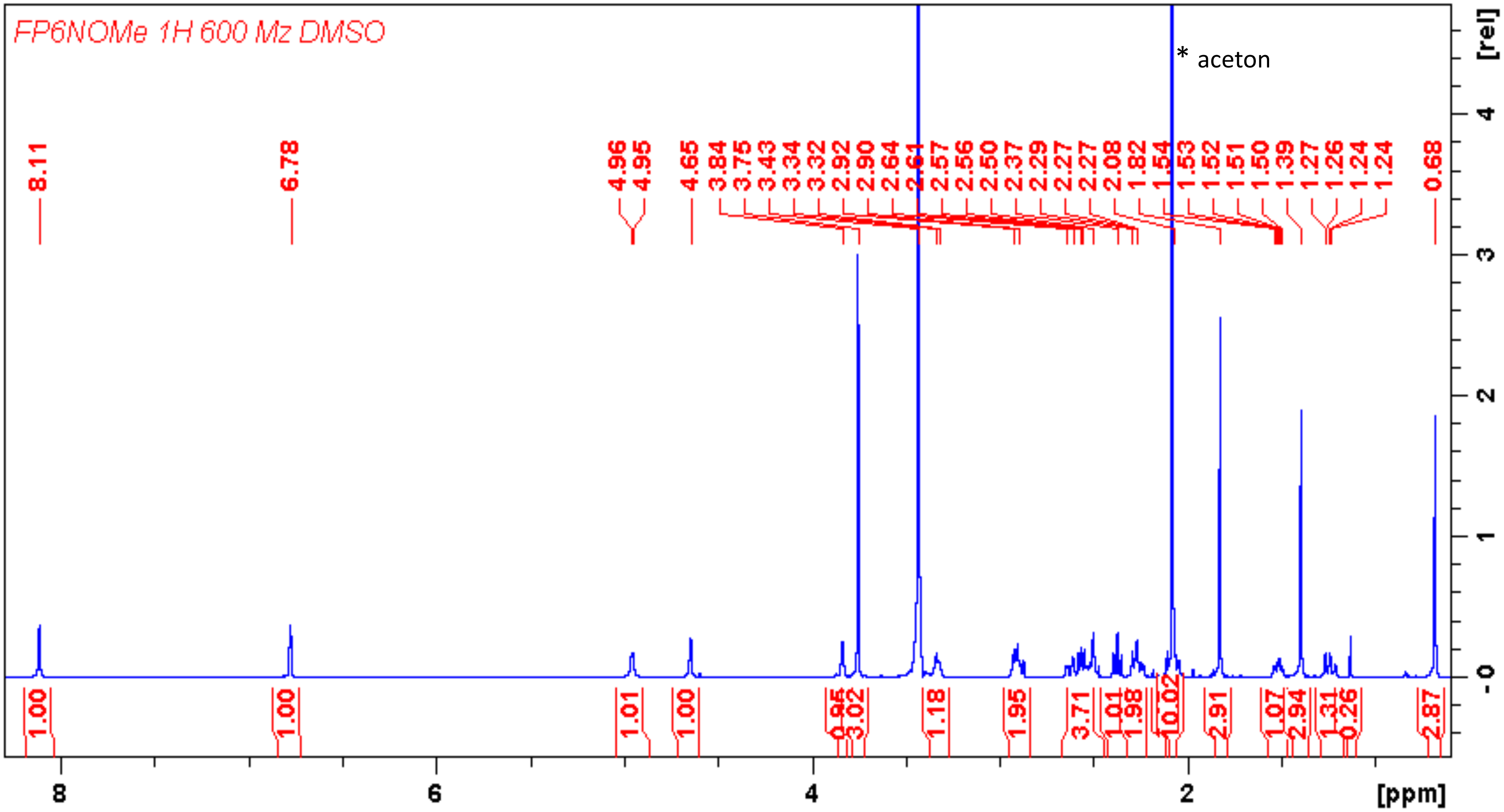

Figure S6. Compound **4** DEPTQ

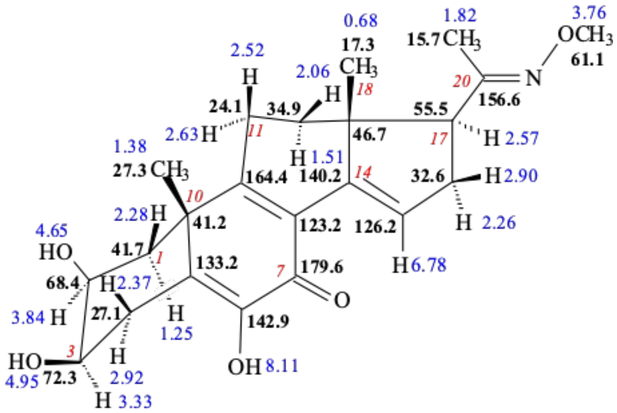

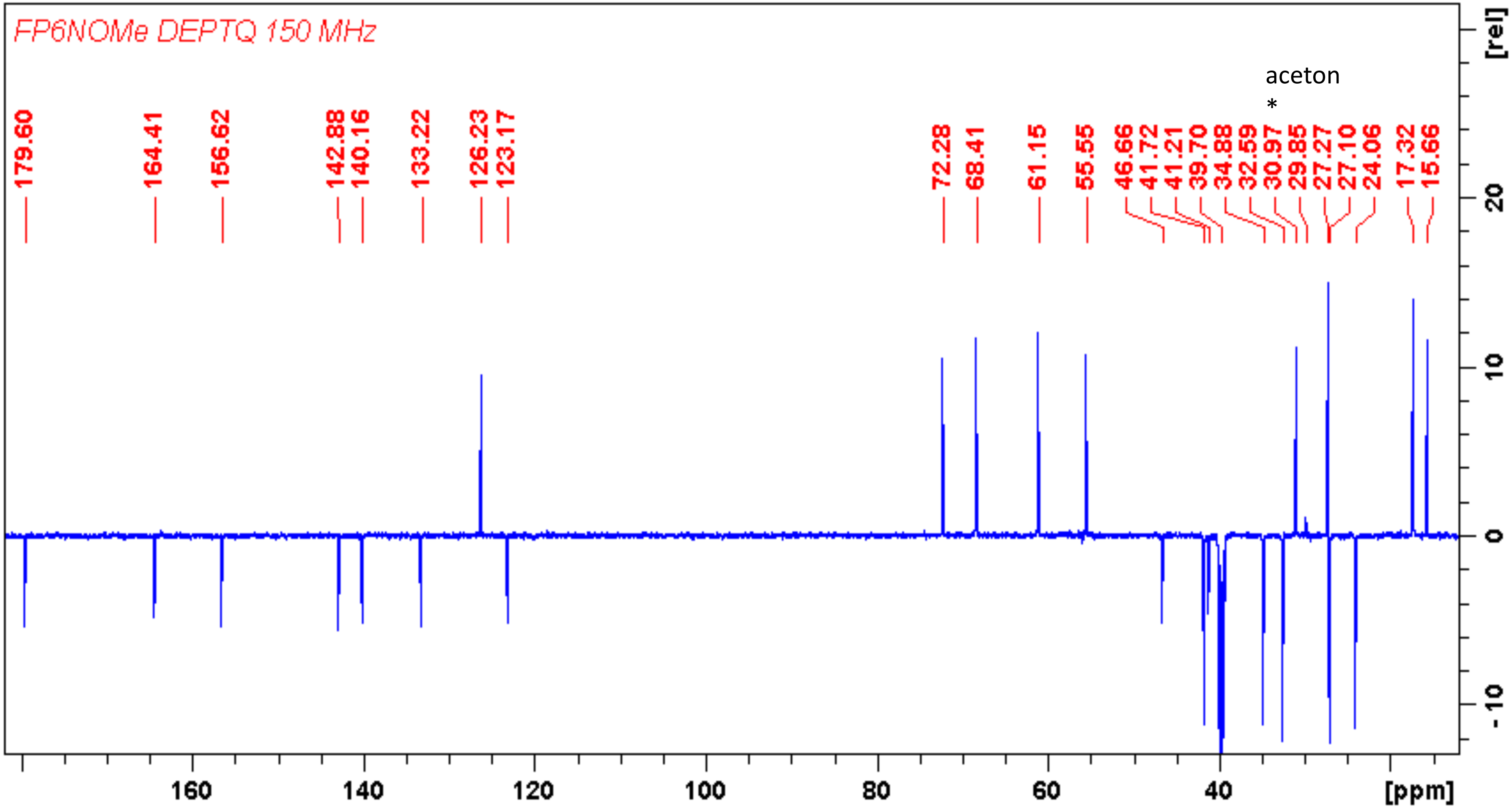

Figure S7. Compound **4** edHSQC

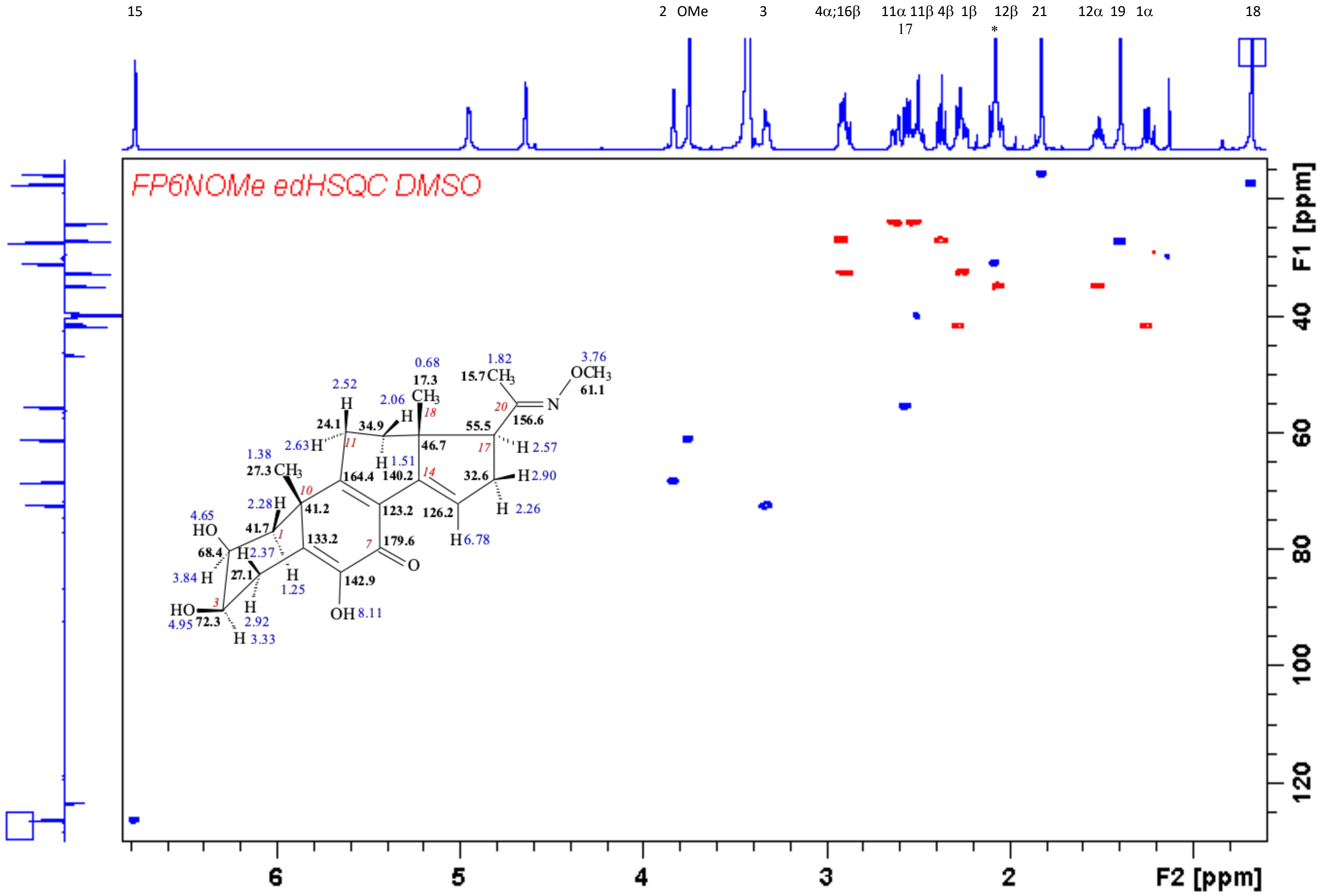

Figure S8. Compound **4** edHSQC section + selROE on CH_3-21

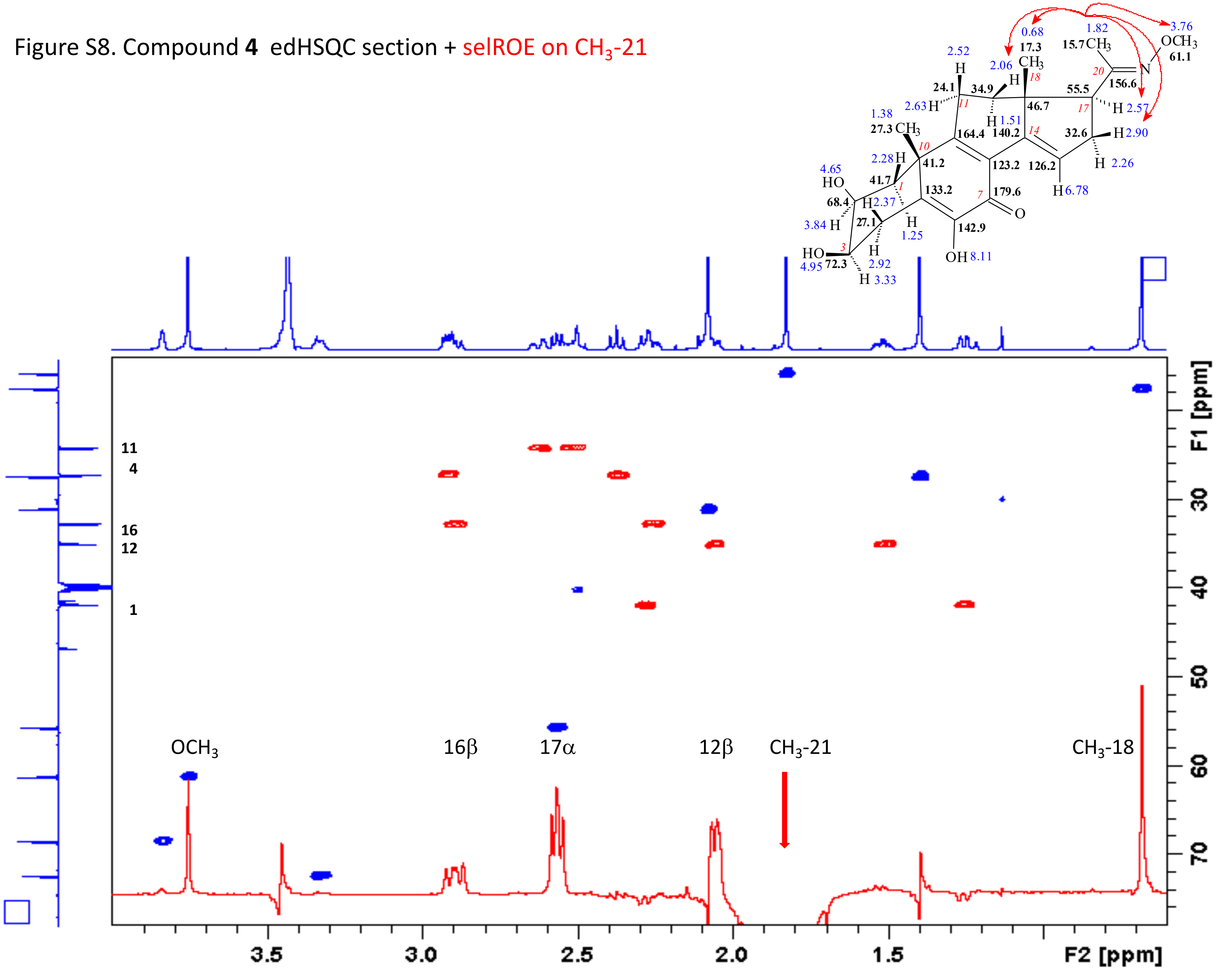

Figure S9. Compound **4**

HMBC CH_3 section

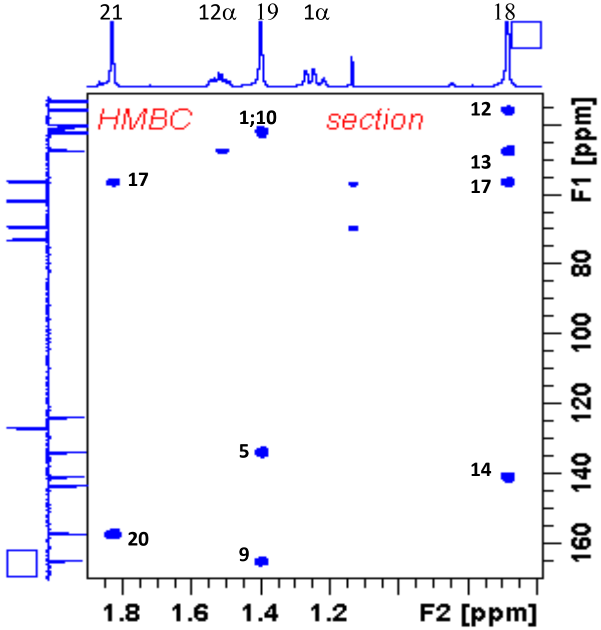

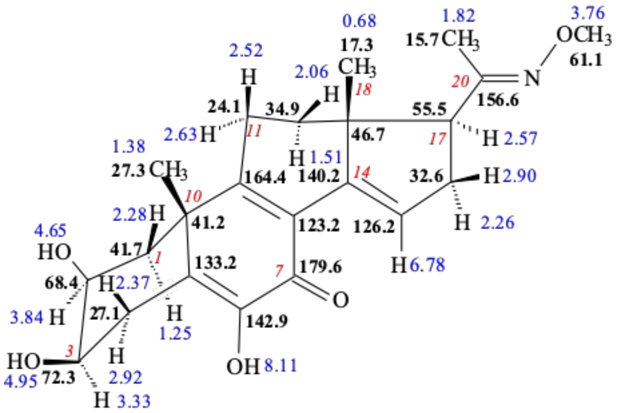

HMBC

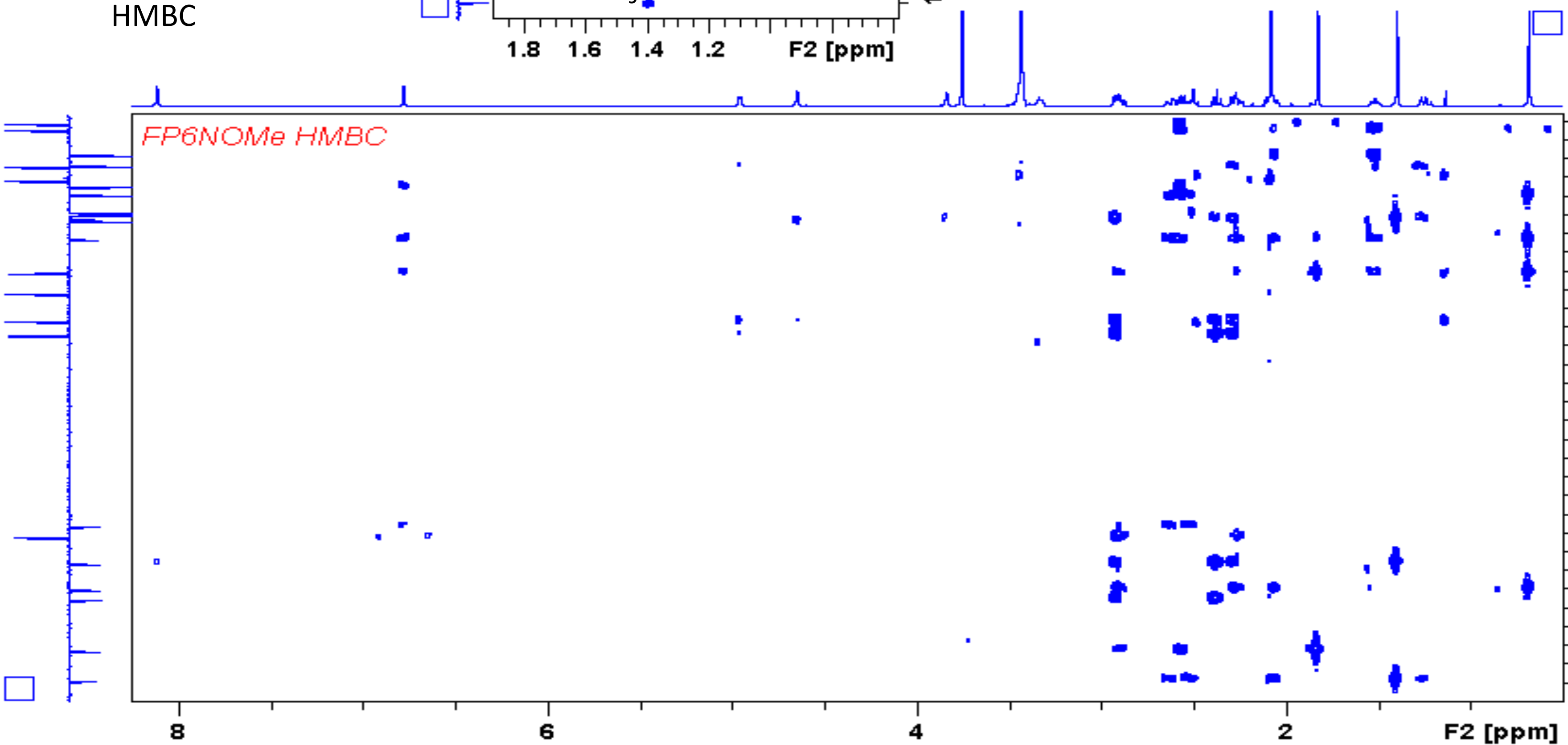

Figure S10. Compound **5** ^{1}H NMR

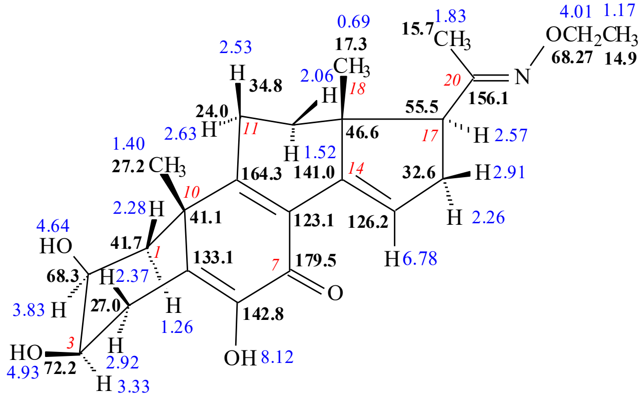

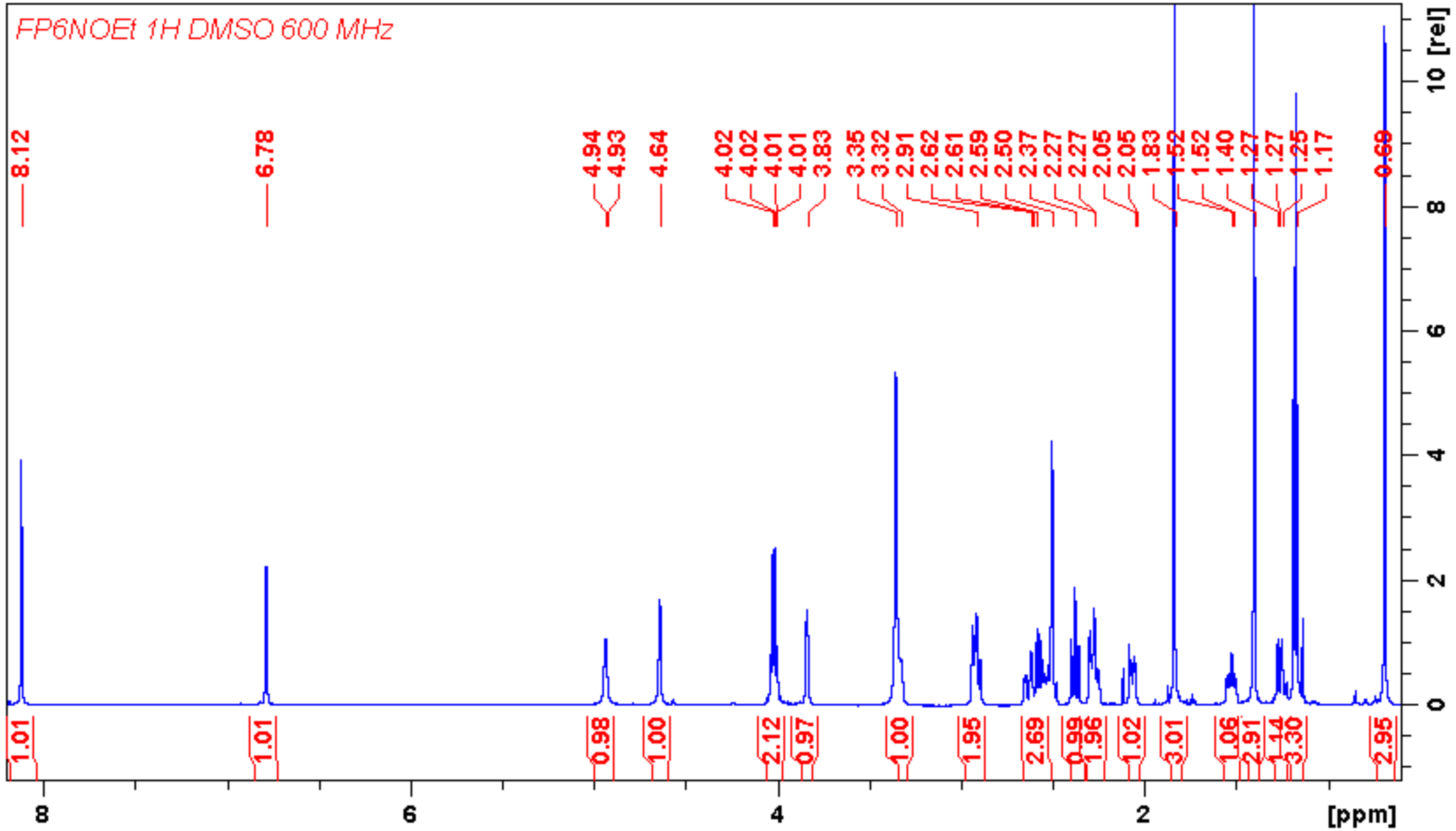

Figure S11. Compound **5** DEPTQ

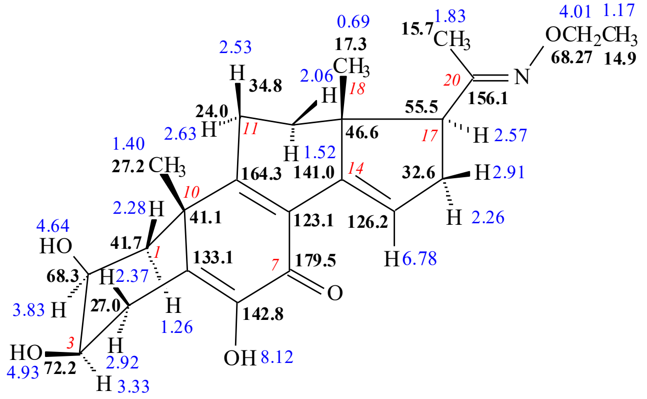

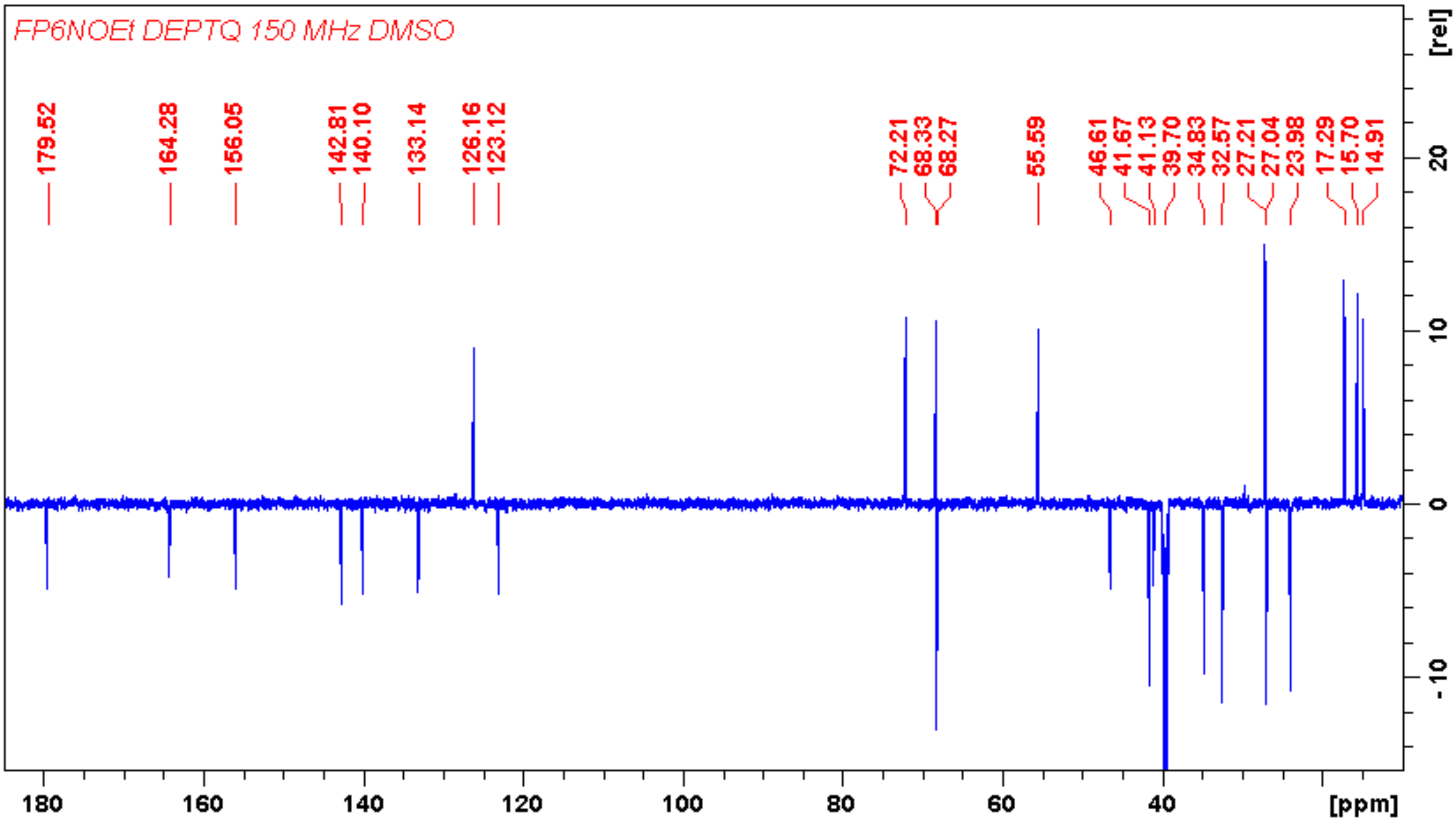

Figure S12. Compound **5** edHSQC

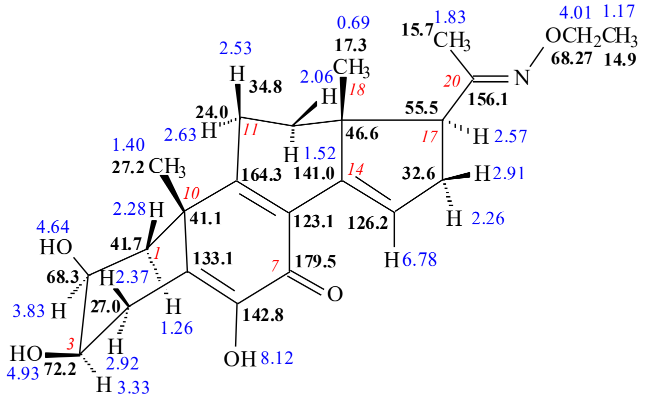

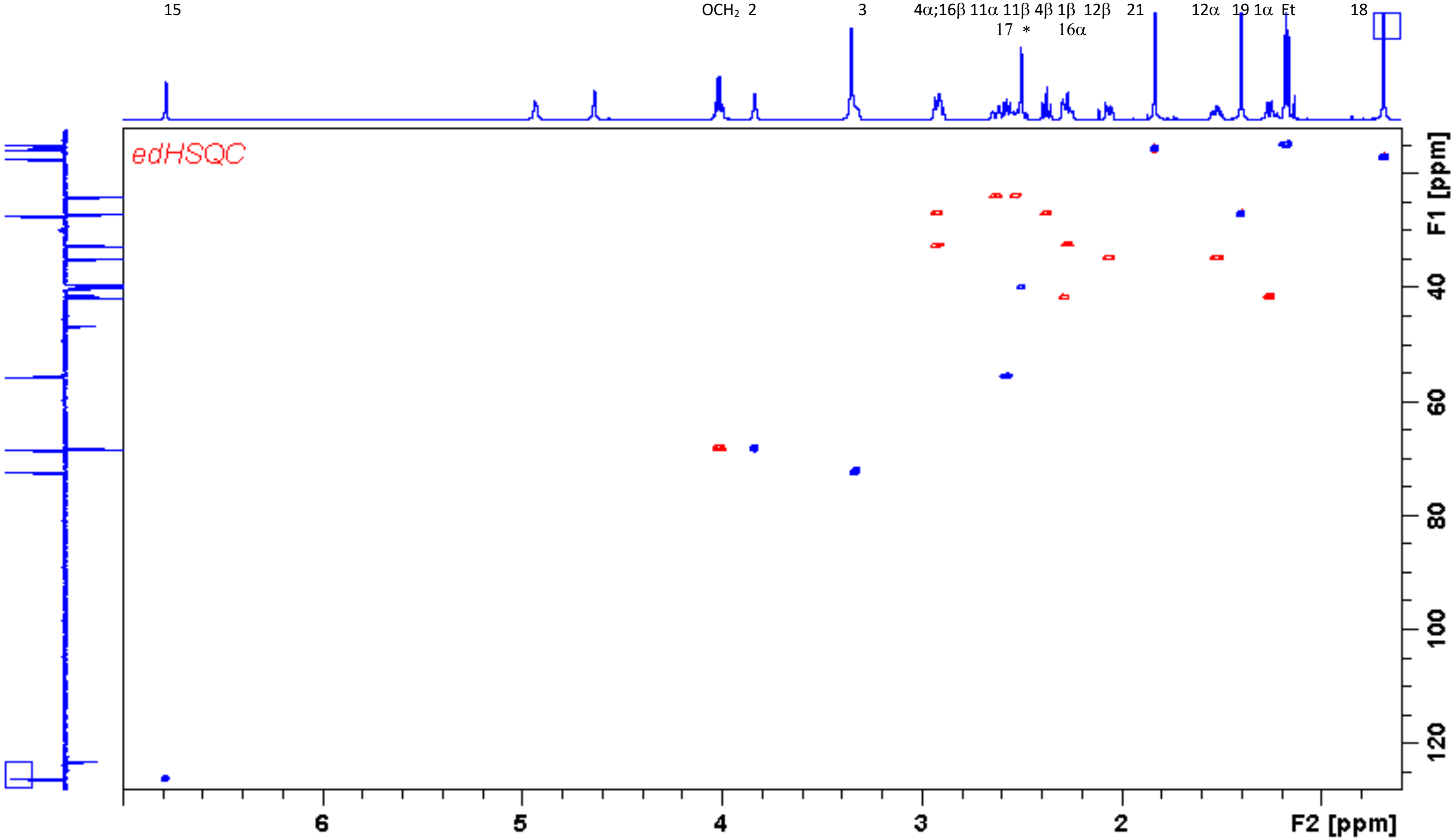

Figure S13. Compound **5** edHSQC section + selROE on CH_3-21

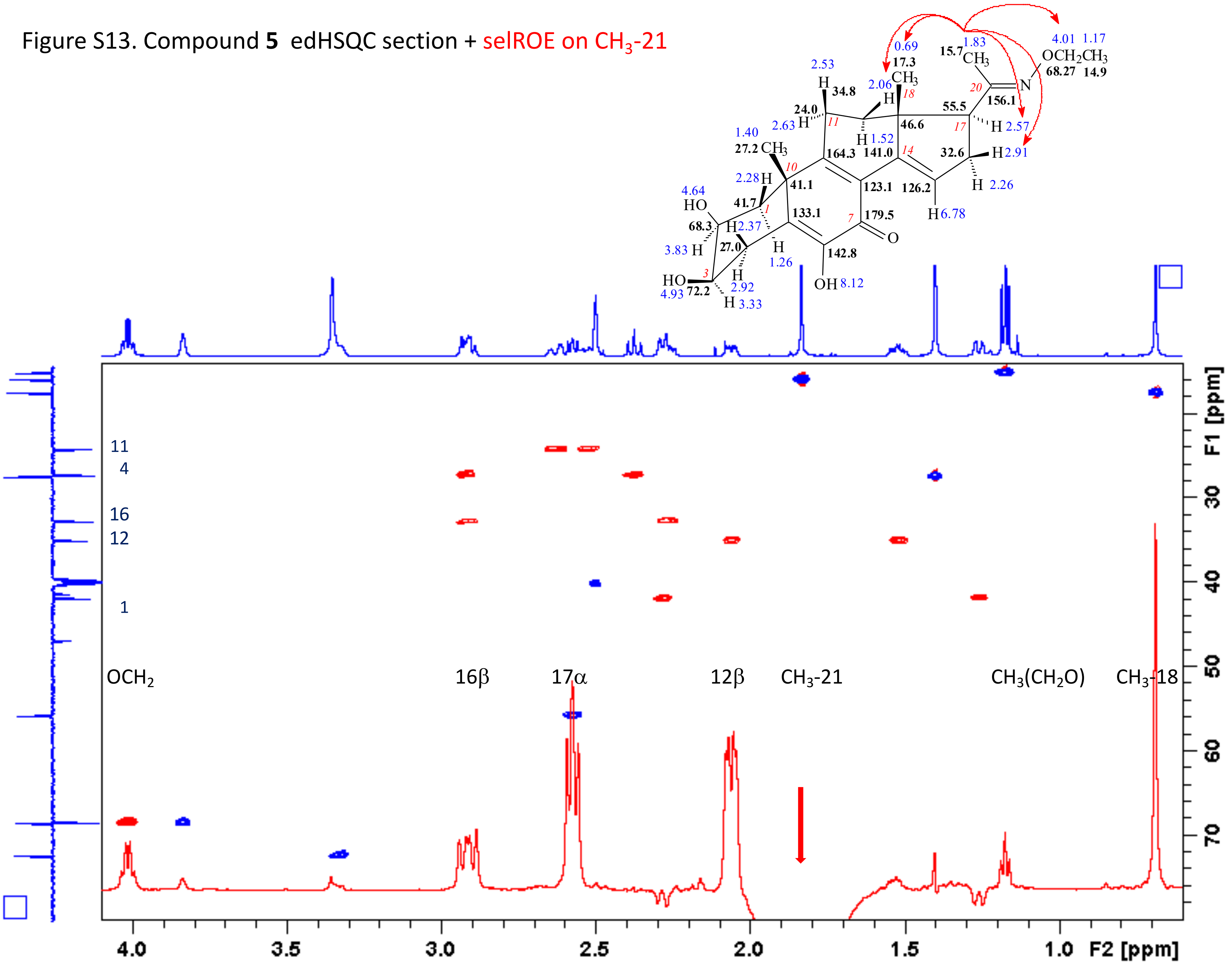

Figure S14. Compound **5** HMBC

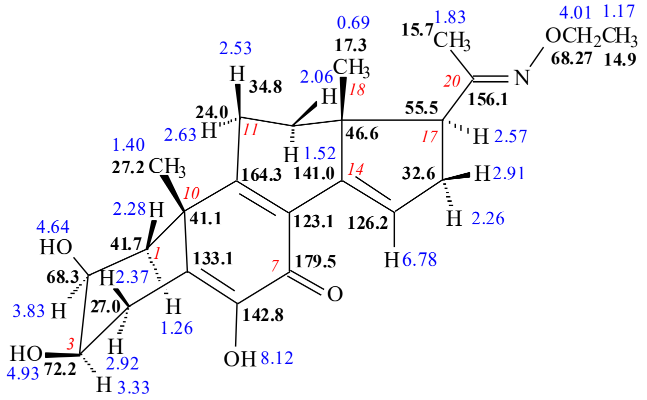

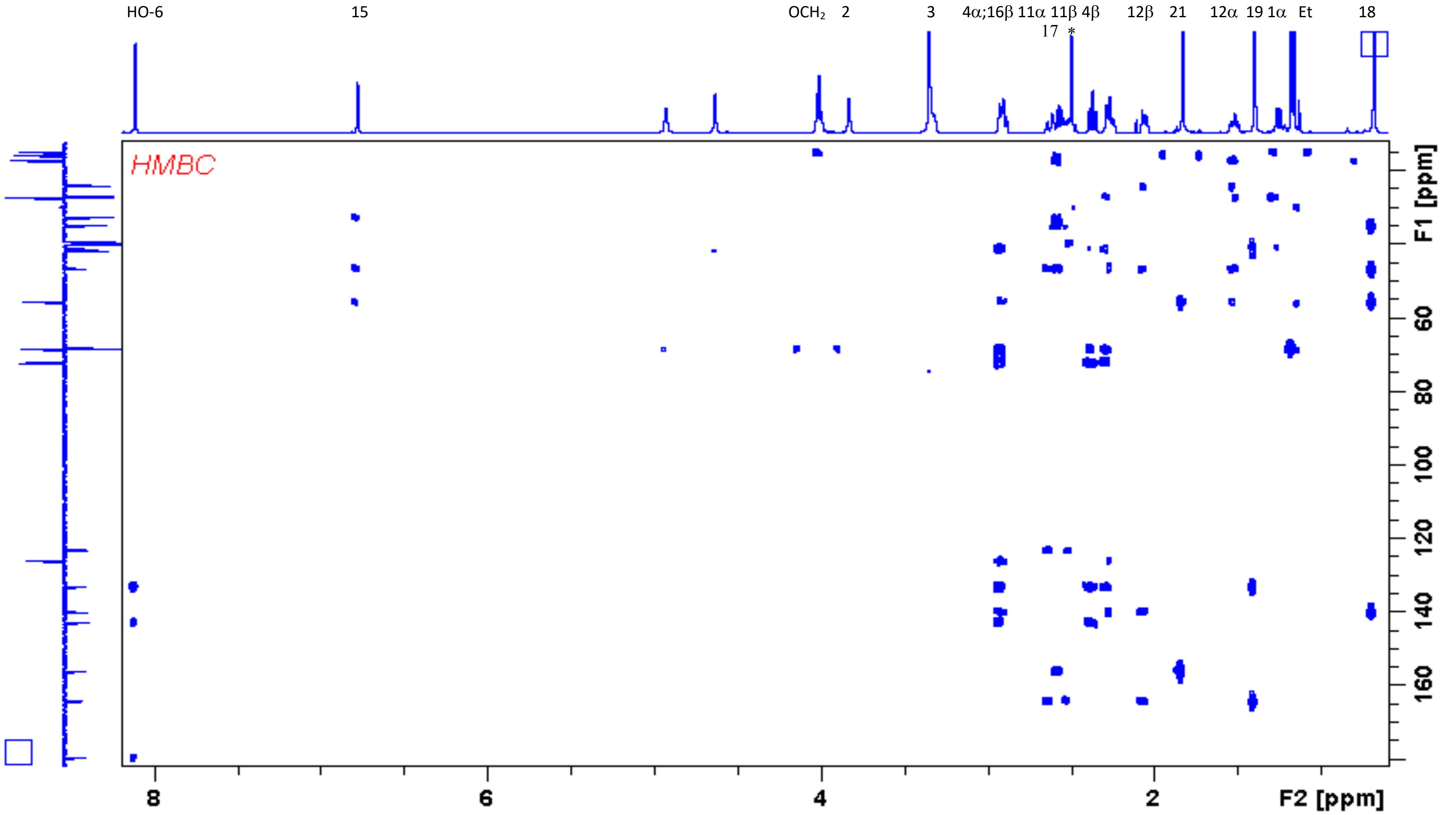

Figure S15. Compound **6** ^{1}H NMR + DEPTQ

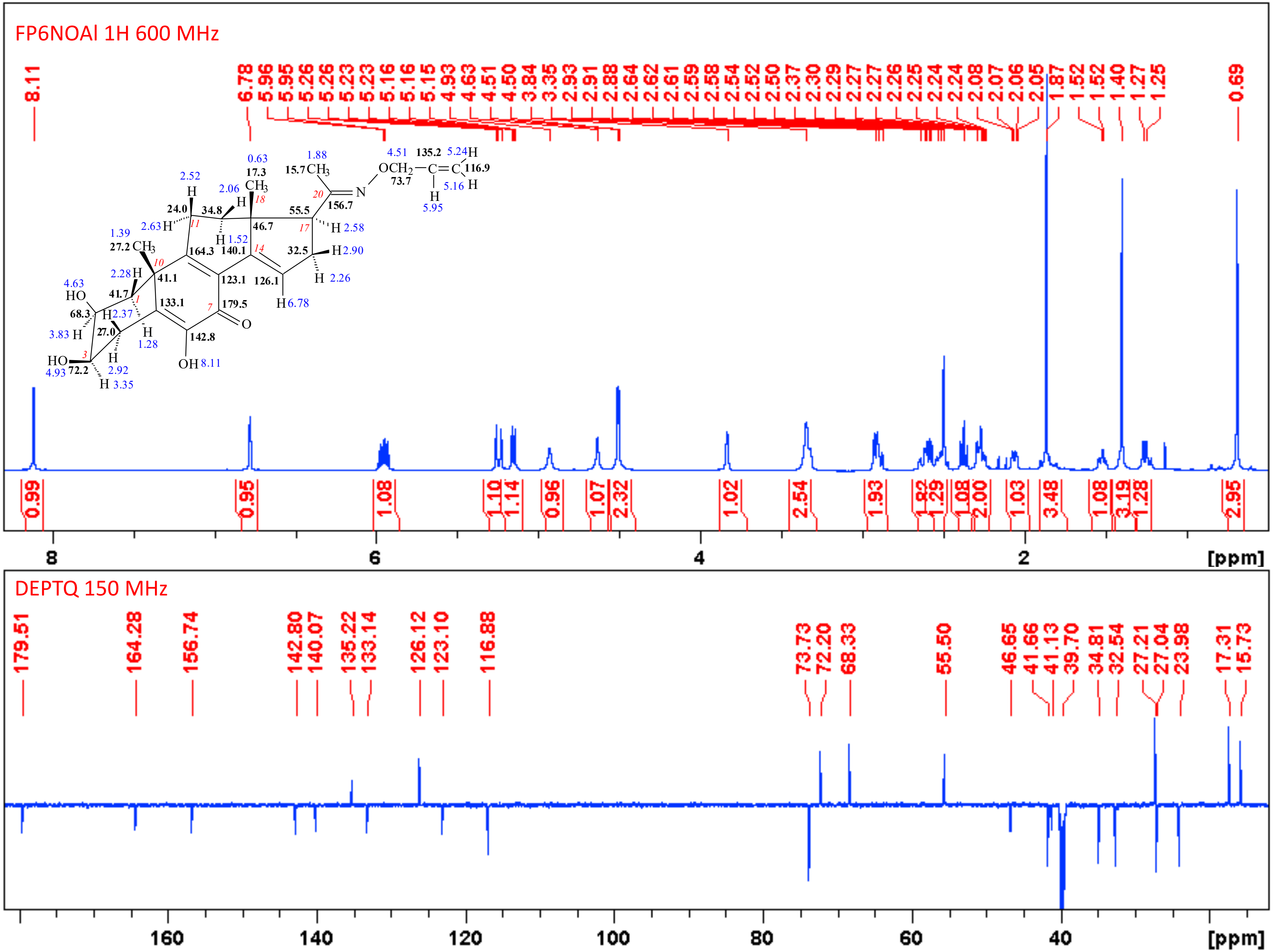

Figure S16. Compound **6** edHSQC

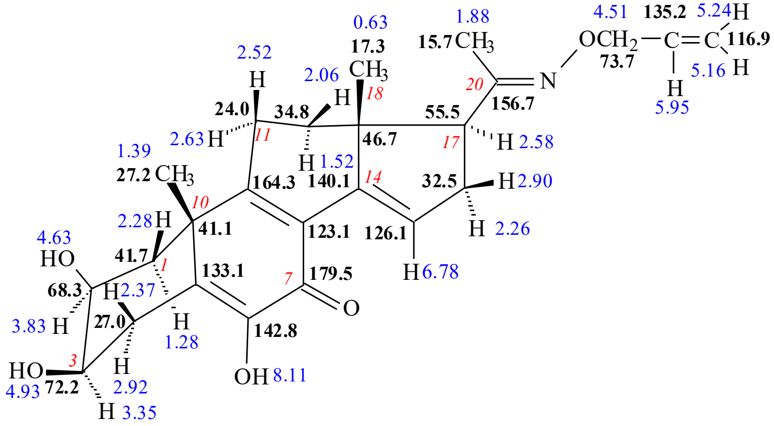

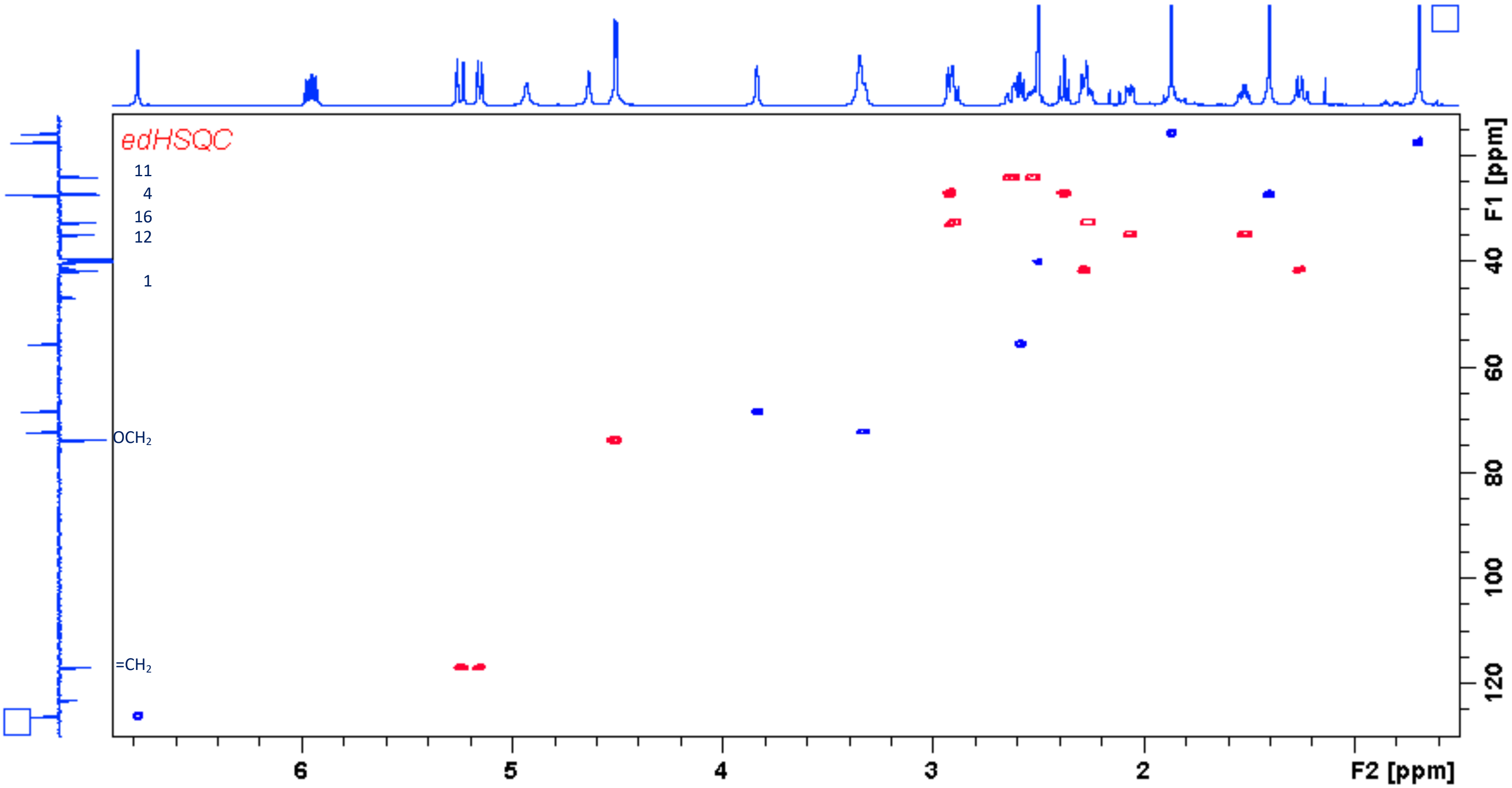

Figure S17. Compound **7** ^{1}H NMR + DEPTQ

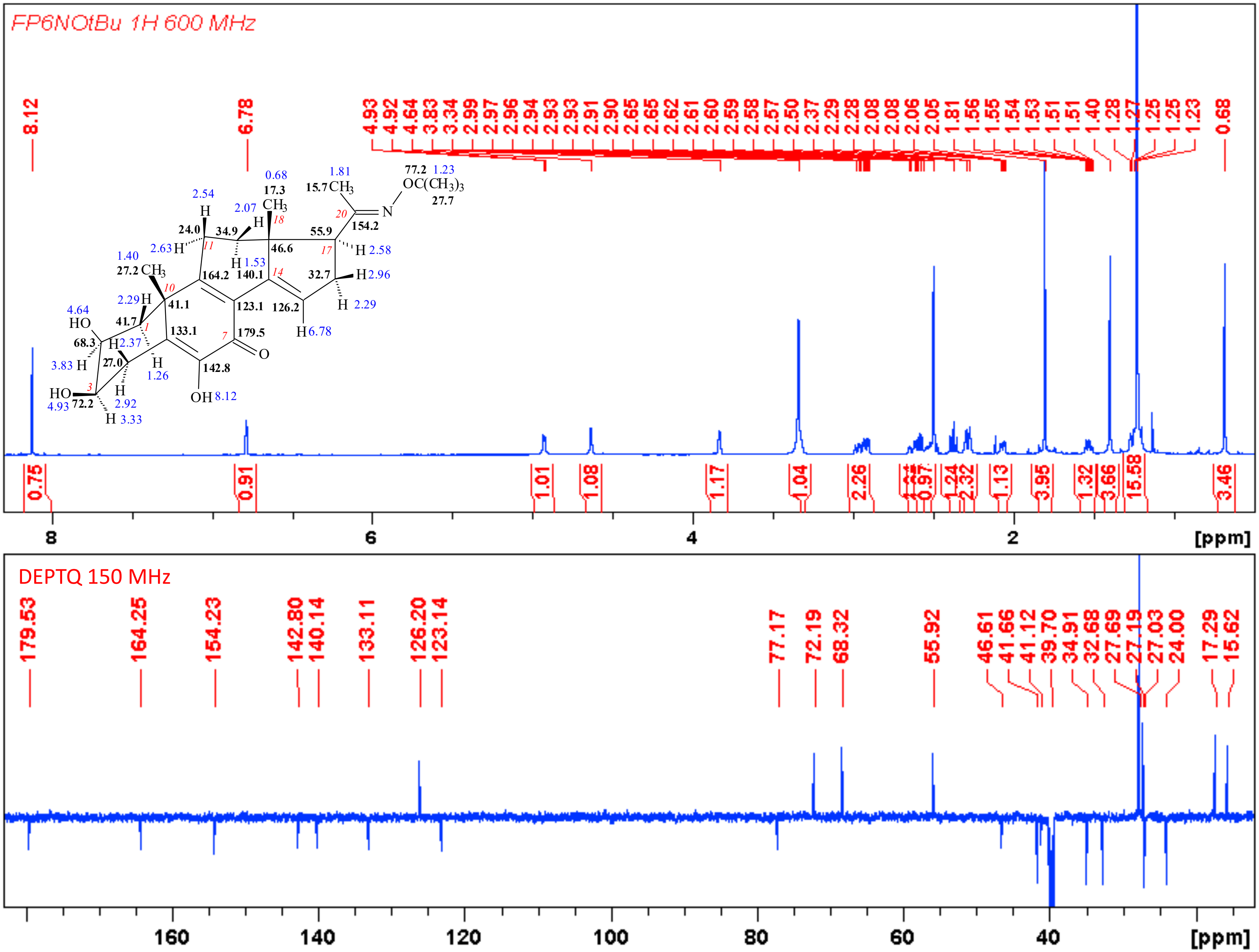

Figure S18. Compound **7** edHSQC

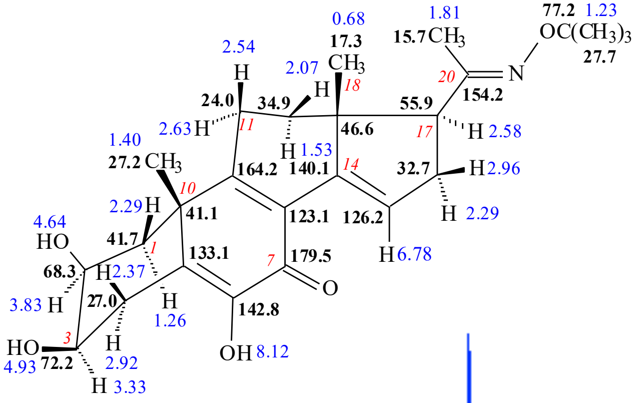

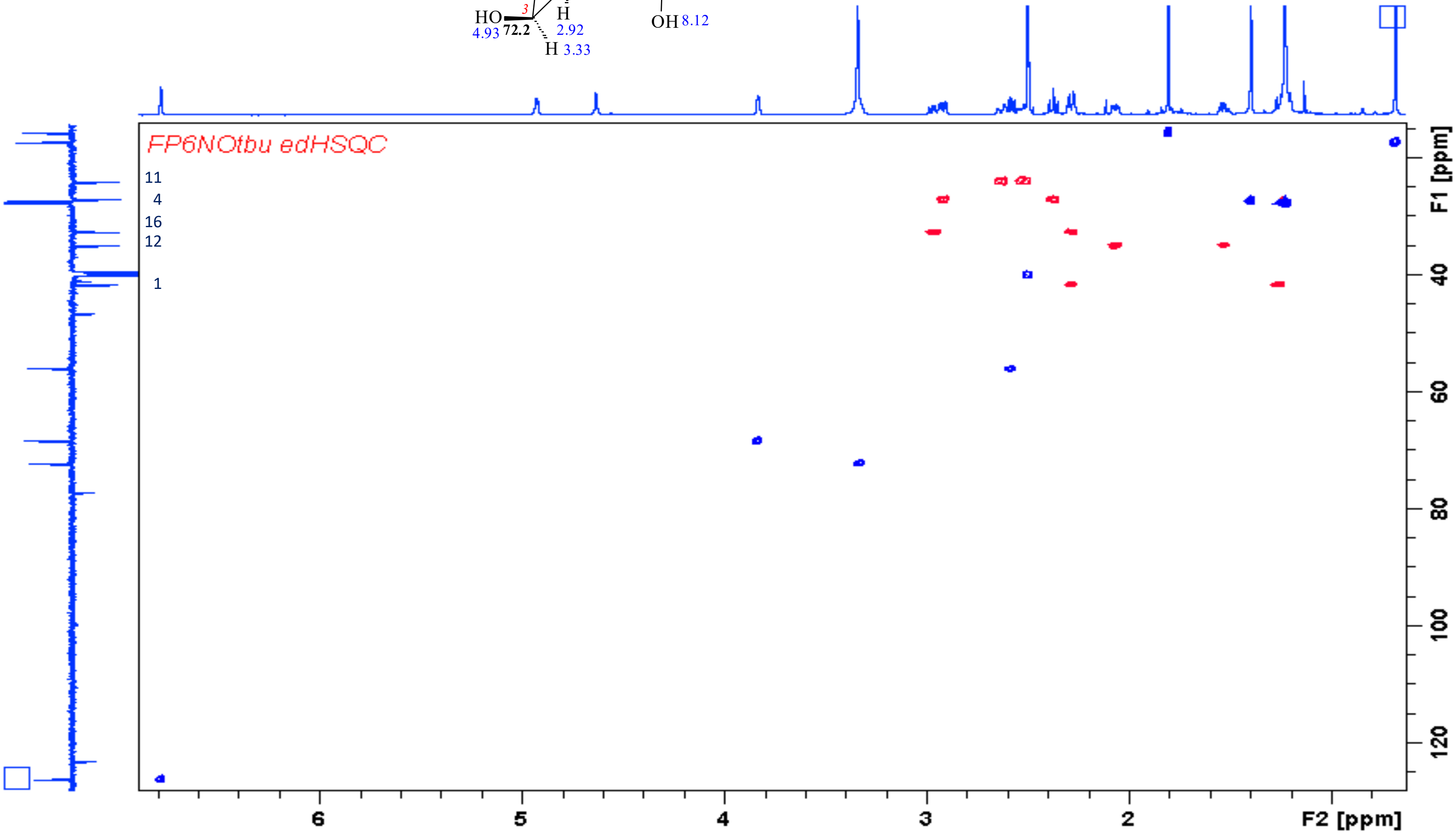

Figure S19. Compound **8** ^{1}H NMR + DEPTQ

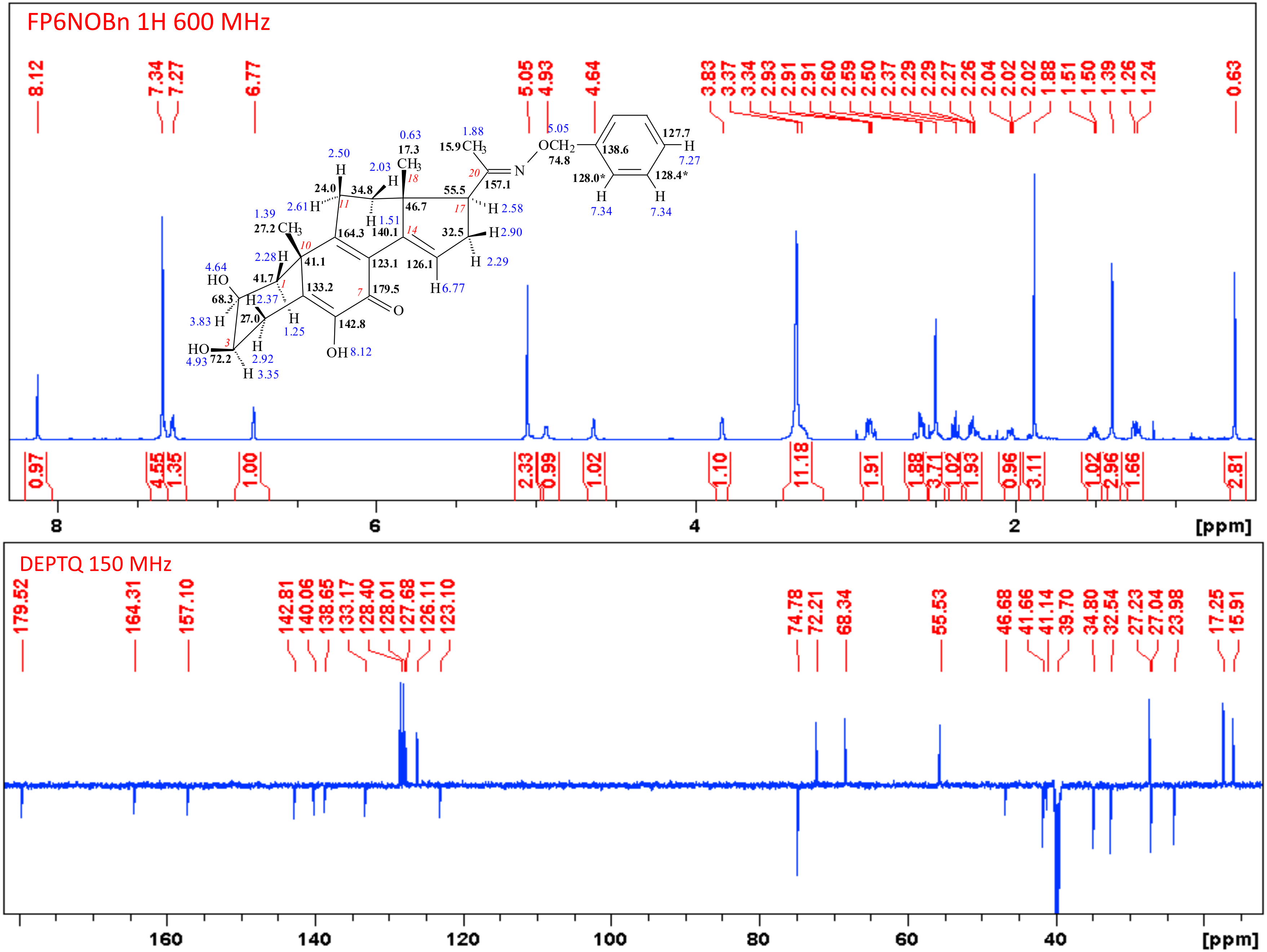

Figure S20. Compound **8** edHSQC

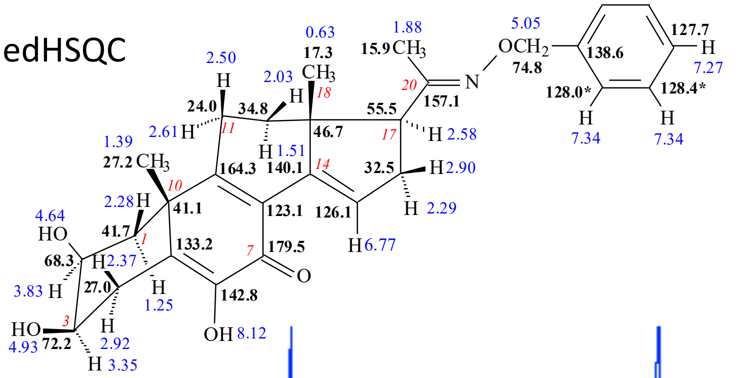

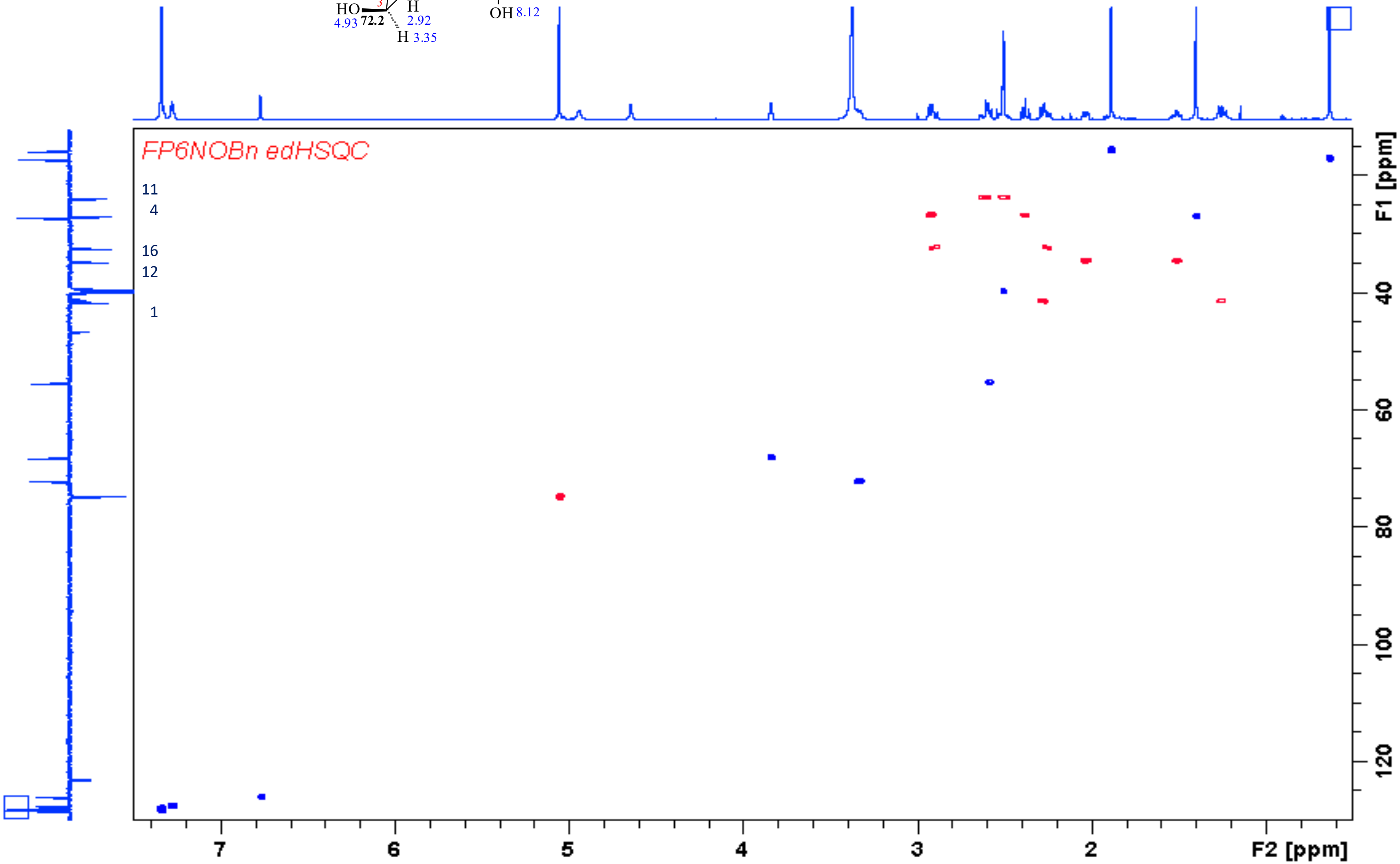

Figure S21. HR-MS spectrum of compound **3** recorded in positive ionization mode.

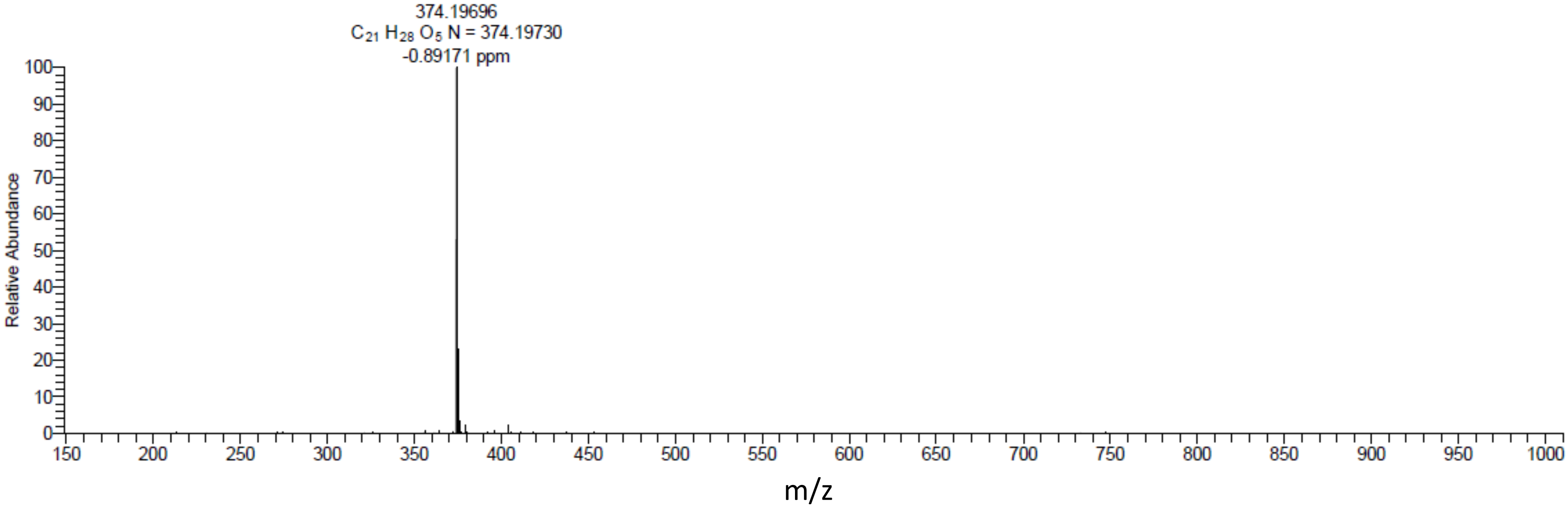

Figure S22. HR-MS spectrum of compound **4** recorded in positive ionization mode.

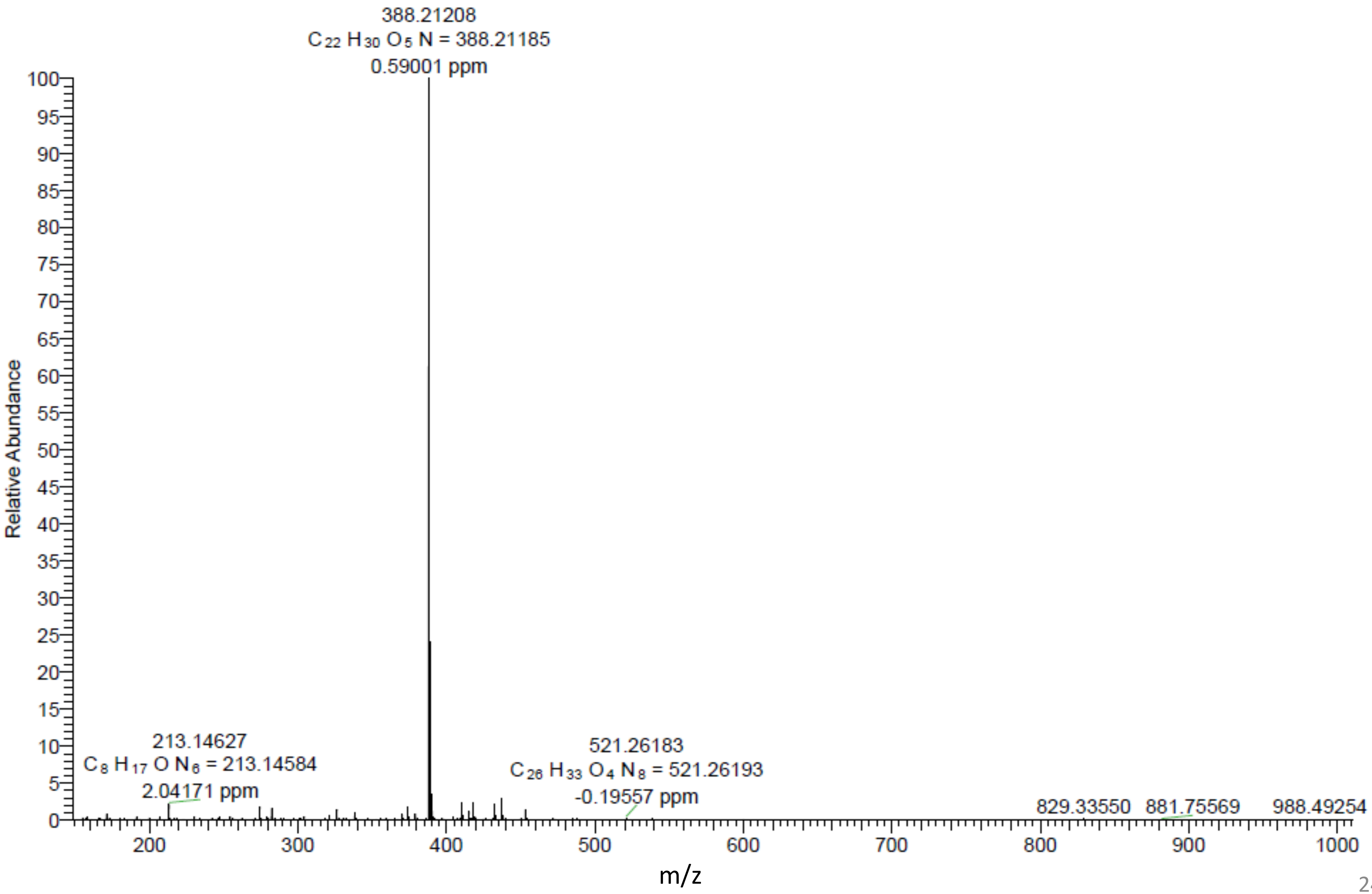

Figure S23. HR-MS spectrum of compound **5** recorded in positive ionization mode.

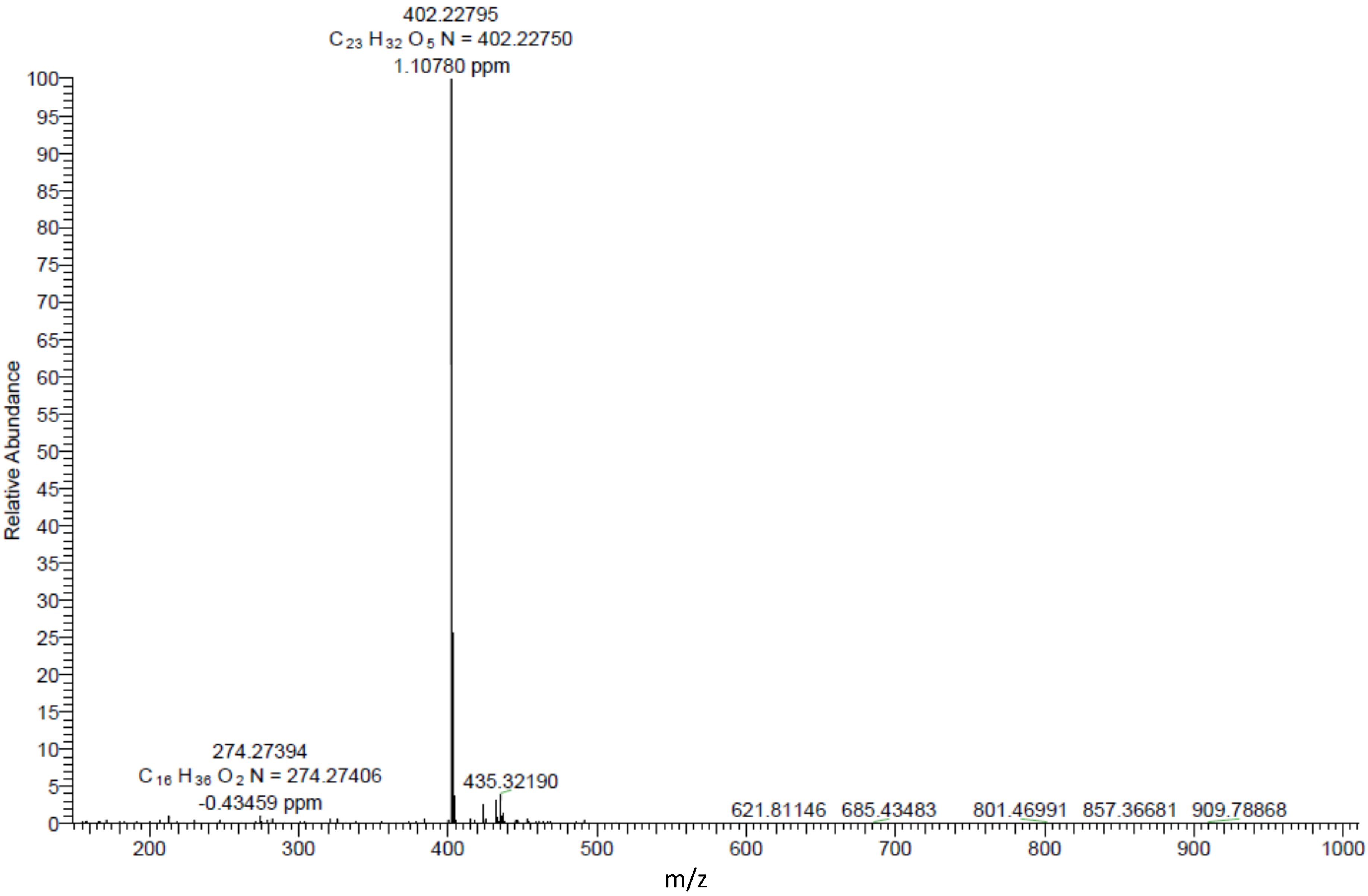

Figure S24. HR-MS spectrum of compound **6** recorded in positive ionization mode.

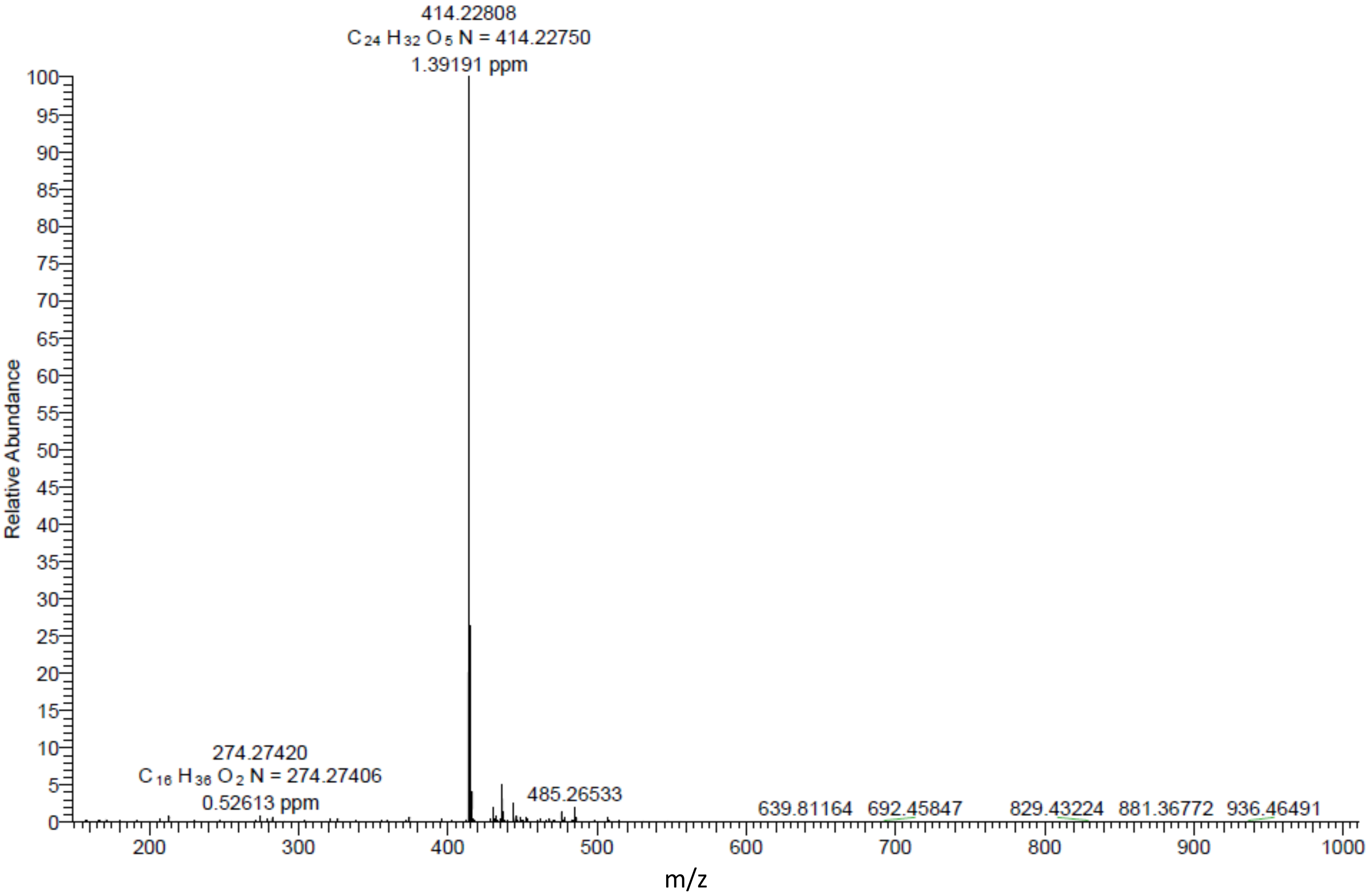

Figure S25. HR-MS spectrum of compound **7** recorded in positive ionization mode.

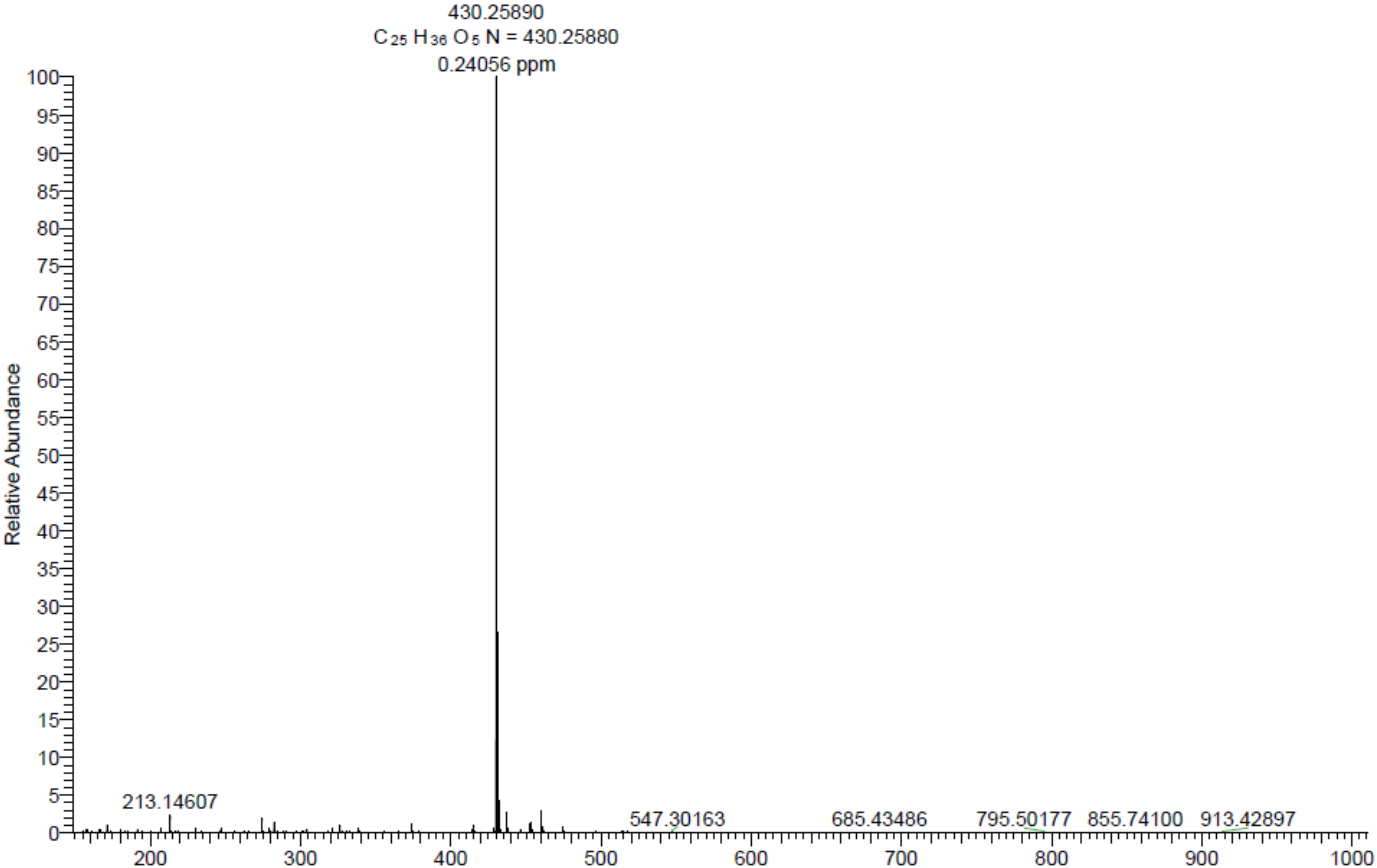

m/z

Figure S26. HR-MS spectrum of compound **8** recorded in positive ionization mode.

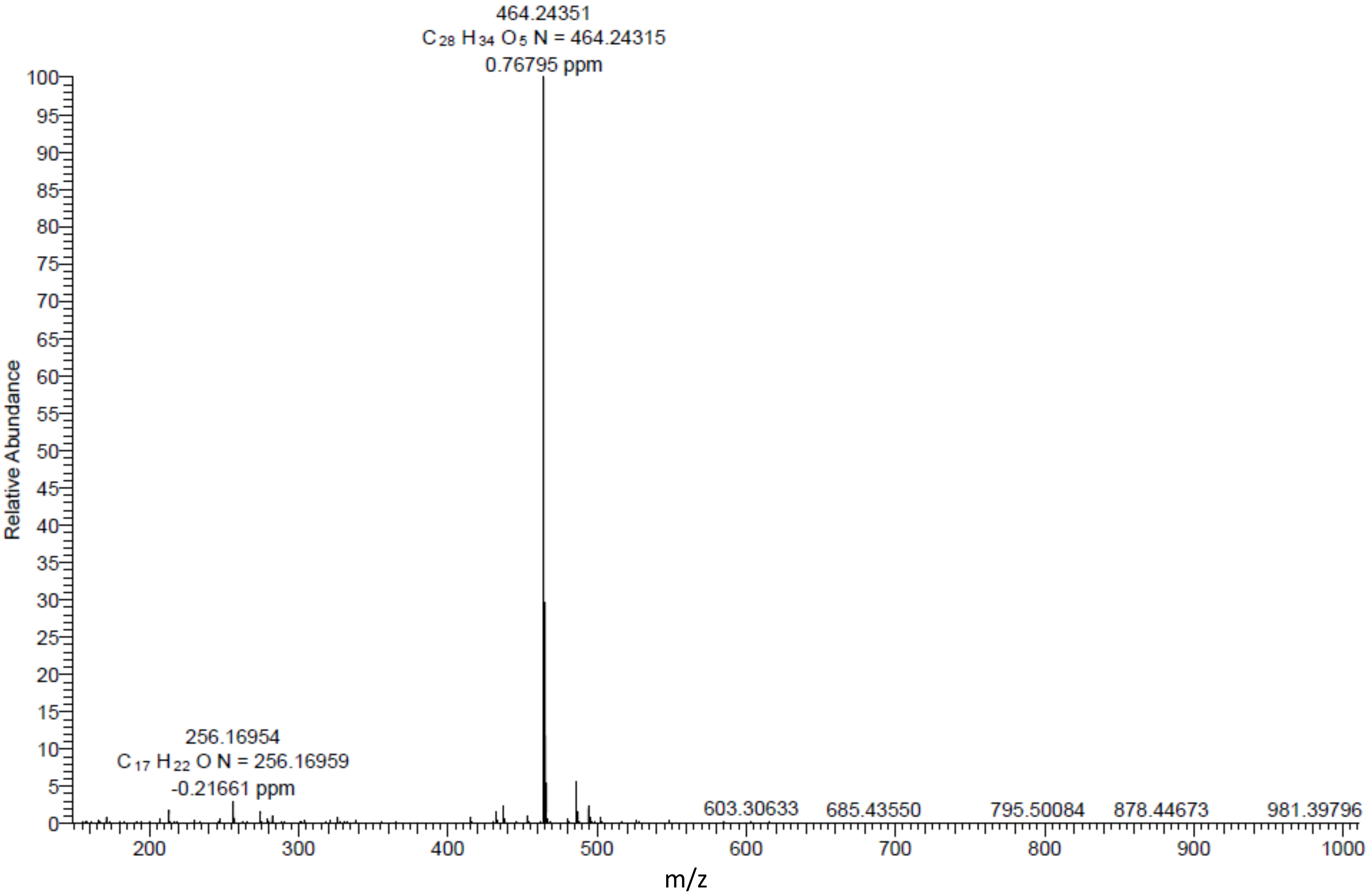

Figure S27. The impact of compounds **2**–**8** on the viability of human brain endothelial cells (hCMEC/D3) was evaluated through impedance-based assays. The cells were treated with compound **2**, **3**, **4**, **5**, **6**, **7** or **8** at concentrations ranging from 0.01 to 10 µM for a duration of 4 hours. The data are presented as the mean ± standard deviation (SD) and were obtained from a minimum of two independent experiments (n = 2−3) with 3−9 technical replicates. Data analysis was performed using one-way analysis of variance (ANOVA) followed by Dunnett's multiple comparisons test. The results were statistically significant with *p < 0.05, ** < 0.01, compared to the control group.

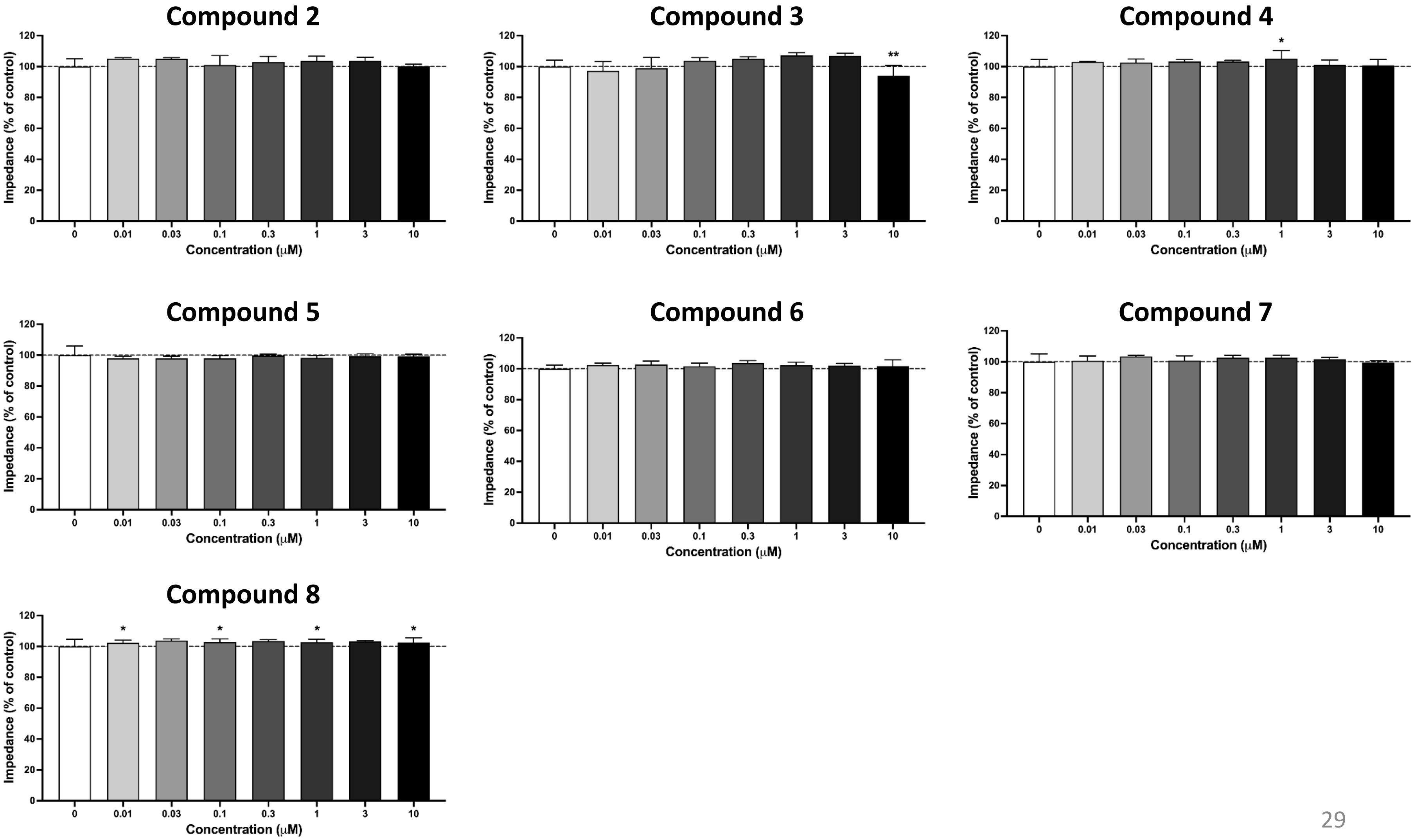

Figure S28. The effects of compounds **4** and **6** at selected concentrations on human brain microvascular endothelial cells (hCMEC/D3).

Impedance-based assays were used to assess cell viability and barrier integrity in the presence of oxidative stress promoted by *tert*-butyl hydroperoxide (tBHP; 350 µM). Cell impedance was monitored for 24 hours. Compounds **4** (3 and 10 µM) and **6** (10 µM) further decreased cell impedance, i.e., sensitized brain endothelial cells to the damaging effect of tBHP-induced oxidative stress.

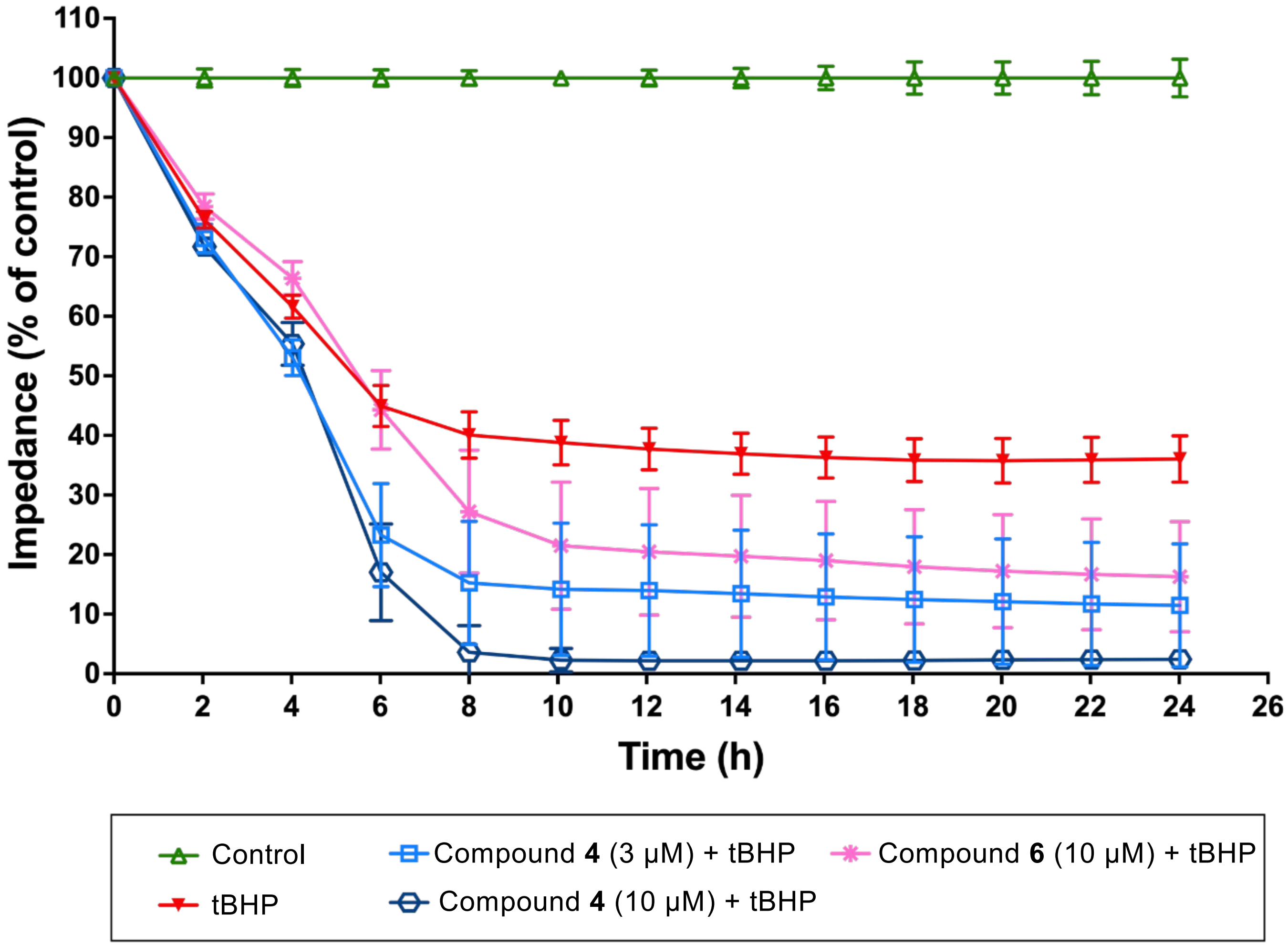

Supplement: S1 File — (PDF) [file pone.0290526.s001.pdf]
